# Navigating Adversity in Older Age: An Integrative Review of Contemporary Resilience Concepts and Measurement Challenges

**DOI:** 10.3390/geriatrics11040111

**Published:** 2026-08-20

**Authors:** Chonlakarn Songsri, Pras Ramluggun, Pimwalunn Aryuwat

**Affiliations:** 1 Faculty of Nursing, Udon Thani Rajabhat University, Udon Thani 41000, Thailand; chonlakarn.so@udru.ac.th; 2 School of Healthcare and Nursing Sciences, Faculty of Health and Wellbeing, Northumbria University, Newcastle NE1 8ST, UK; pras.ramluggun@northumbria.ac.uk; 3 Boromarajonani College of Nursing Udonthani, Faculty of Nursing, Praboromarajchanok Institute, Udon Thani 41330, Thailand

**Keywords:** aging, interventions, older adults, resilience, well-being

## Abstract

**Background/Objectives**: Ageing populations face complex challenges, yet many older adults maintain well-being despite adversity. Understanding resilience offers critical direction for health promotion. This integrative review takes a conceptual and methodological approach to resilience in older adults, with clear implications for geriatric care. The review addresses: (1) how resilience is conceptualised and measured; (2) individual, social, and contextual factors linked to resilience; and (3) the effectiveness of interventions that aim to promote resilience. **Methods**: Following Whittemore and Knafl’s framework, a systematic search of six databases (PubMed, CINAHL, MEDLINE, Scopus, Web of Science, and Cochrane CENTRAL; 2016–2025) identified 42 empirical studies across 18 countries of diverse designs. Quality was assessed using the Mixed Methods Appraisal Tool, and thematic analysis identified five convergent themes. **Results**: Most studies explicitly defined resilience using trait-, process-, or outcome-based frameworks and employed validated instruments, most commonly the CD-RISC. Self-efficacy, spirituality, sense of coherence, and gratitude were consistently associated with resilience. Family relationships, social networks, and community participation were important relational correlates. Health status, education, economic security, and digital literacy were key contextual factors. Six longitudinal studies linked higher resilience to reduced mortality, slower cognitive decline, and attenuated frailty progression. Seven intervention studies, including five randomised controlled trials, supported physical activity, mindfulness, positive thinking training, Tai Chi, and technology-supported exercise as promising intervention modalities, though one web-based programme yielded null findings. **Conclusions**: Resilience in older adults is a multifaceted and potentially modifiable construct. Growing longitudinal evidence extends its relevance beyond psychological well-being to physical health outcomes, though cross-sectional designs predominate and limit causal inference. Measurement heterogeneity across more than twenty instruments underscores the need for international consensus and culturally valid tools. Larger longitudinal studies and adequately powered trials are needed to guide definitive practice and policy for ageing populations worldwide.

## 1. Introduction

Ageing presents a multifaceted global challenge, placing particular strain on healthcare systems as the burden of chronic disease and long-term care needs continues to rise [1]. The older population aged 60 years and above will reach approximately 2.1 billion by 2050, with those aged 80 and older representing the fastest-growing demographic segment [2]. This demographic shift carries important implications for individual health outcomes and public health infrastructure, as advancing age is commonly accompanied by multiple chronic conditions, functional decline, social losses, and psychological stressors [3,4]. Subjective well-being and emotional regulation, however, often improve from middle age into early old age even as physical health declines, a pattern described as the U-shaped curve of happiness [5]. Ageing is also associated with greater lived experience, self-acceptance, and a sense of mastery [6], qualities that are closely linked to resilience.

Resilience, defined as the capacity to maintain or regain well-being despite exposure to adversity, has emerged as a central framework for understanding successful ageing and informing health promotion interventions [6,7,8]. Foundational theoretical and empirical work by Rutter [9], Staudinger et al. [10], and Infurna and Luthar [11] established resilience as a core construct in lifespan developmental science. Existential concerns such as death anxiety have also been shown to reduce resilience in older adults, with attachment style, reflecting the capacity to seek and benefit from close relationships, moderating this association [12]. Cognitive challenges such as mild cognitive impairment also intersect with resilience in older adults; negative affect, including depression, anxiety, and stress, has been shown to challenge psychological resilience, whereas positive emotions and a sense of accomplishment remain relatively preserved in early-stage, suggesting that resilience is differentially shaped by the degree of cognitive decline [13]. Resilience is also linked to personal qualities, including optimism and self-confidence, as well as to social and environmental influences, including family relationships, social networks, and spiritual engagement [14,15,16]. Contextual factors such as health status, education, and socioeconomic resources also influence resilience outcomes [17]. In addition, structured interventions, including exercise programs, mindfulness approaches, and nature-based therapies, can enhance resilience [18].

Several recent reviews have examined protective factors in successful ageing [19,20], yet fewer have linked resilience concepts and measurement directly to longitudinal health outcomes and intervention evidence within a single synthesis. This review contributes to the literature in three ways: it brings together conceptual, predictor, and intervention evidence within one framework; it incorporates recent longitudinal studies linking resilience to mortality, cognitive decline, and frailty; and it offers practice and policy directions specific to geriatric care. The review addresses three aims: (1) how resilience is conceptualised and measured; (2) individual, social, and contextual factors linked to resilience; and (3) the effectiveness of interventions that aim to promote resilience. For the purposes of this review, conceptualisation refers to the explicit or implicit theoretical framing of resilience adopted by each study, such as resilience as a trait, process, outcome, or trajectory, whilst measurement refers to the operational instrument used to quantify it. Two research questions guided the review: How is resilience conceptualised, defined, and manifested in the context of ageing? What types of interventions have proven efficacy in enhancing resilience in older adult populations?

## 2. Materials and Methods

We conducted an integrative review of empirical literature to analyse this complex topic, as this methodology is designed to synthesise diverse forms of evidence (e.g., qualitative, quantitative) alongside theoretical literature. Whilst integrative reviews can, in principle, synthesise empirical and theoretical sources [21], this review was deliberately restricted to empirical studies to ensure that conclusions about resilience conceptualisation, predictors, and interventions were grounded in primary data rather than secondary theoretical commentary. Theoretical and review papers were therefore excluded, though key theoretical frameworks are drawn upon in the Introduction and Discussion to contextualise empirical findings.

This integrative review followed Whittemore and Knafl’s methodological framework, comprising: (1) problem identification, (2) literature search, (3) data evaluation, (4) data analysis, and (5) presentation. The problem identification stage has been addressed in the introduction [21]. A systematic search of six databases (PubMed, CINAHL, MEDLINE, Scopus, Web of Science, and Cochrane CENTRAL) was conducted (see Section 2.1 for full details, including database interfaces, subject headings, and search fields). Complete per-database search strategies, the PRISMA checklist, and the full data extraction table are provided as Supplementary Materials.

The review addressed two complementary research questions: how resilience is conceptualised and measured and what interventions enhance it, which are both served by the same search strategy and inclusion criteria. Studies were included if they: measured resilience as an outcome, predictor, or mediating/moderating variable; involved adults aged 60 years and above only, as the entire study sample; and were published in English as peer-reviewed empirical research.

Studies were excluded if they were: reviews or theoretical papers; included participants under 60 years of age; published in a language other than English; or focused exclusively on populations with severe mental illness or disability. Two independent reviewers screened records; disagreements were resolved by a third reviewer. Data extraction captured study characteristics, demographics, resilience definitions, instruments, and findings. Quality was assessed using the Mixed Methods Appraisal Tool (MMAT) [22]. Narrative synthesis with thematic analysis was employed [23].

### 2.1. Search Strategy and Eligibility Criteria

Six databases were searched: PubMed, CINAHL, and MEDLINE were searched using the original search strategy for consistency with the initial submission, and Scopus, Web of Science, and Cochrane CENTRAL were added following peer review, using a refined strategy with truncation (e.g., resilien*, elder*, senior*, geriatric*) to improve sensitivity, as recommended by the assigned research librarian. The core search string combined: (resilience OR “bounce back” OR “psychological resilience” OR “stress adaptation” OR hardiness) AND (“older adult” OR “elder” OR “aged” OR “ageing” OR “senior” OR “geriatric”), adapted to each database’s syntax and fields (title/abstract/keywords in Scopus; Topic in Web of Science; title/abstract in CINAHL; Abstract/Keyword in Cochrane CENTRAL). Six databases were chosen to provide strong coverage of nursing, allied health, biomedical, and broader interdisciplinary research relevant to this review, with Scopus and Web of Science added specifically to improve coverage beyond biomedical databases. PubMed and MEDLINE index substantially overlapping biomedical content, which accounts for a considerable proportion of the duplicate records identified during screening; this overlap was accepted as a trade-off for ensuring comprehensive biomedical coverage, and duplicates were removed prior to title/abstract screening. Other databases commonly used in resilience and gerontological research, including APA PsycINFO, APA PsycArticles, AgeLine, and Embase, were not searched, as they were not available through the authors’ institutional subscriptions; this is acknowledged as a limitation in Section 4.5. Full per-database search strategies, fields, limits, and execution dates are reported in Supplementary Section S1. The search was limited to English-language publications for practical reasons of translation capacity. We recognise that both the database restriction and the language restriction may have excluded relevant studies, particularly from non-English-speaking countries where cultural views of resilience may differ. This is discussed further as a limitation in Section 4.5.

**Figure 1:**
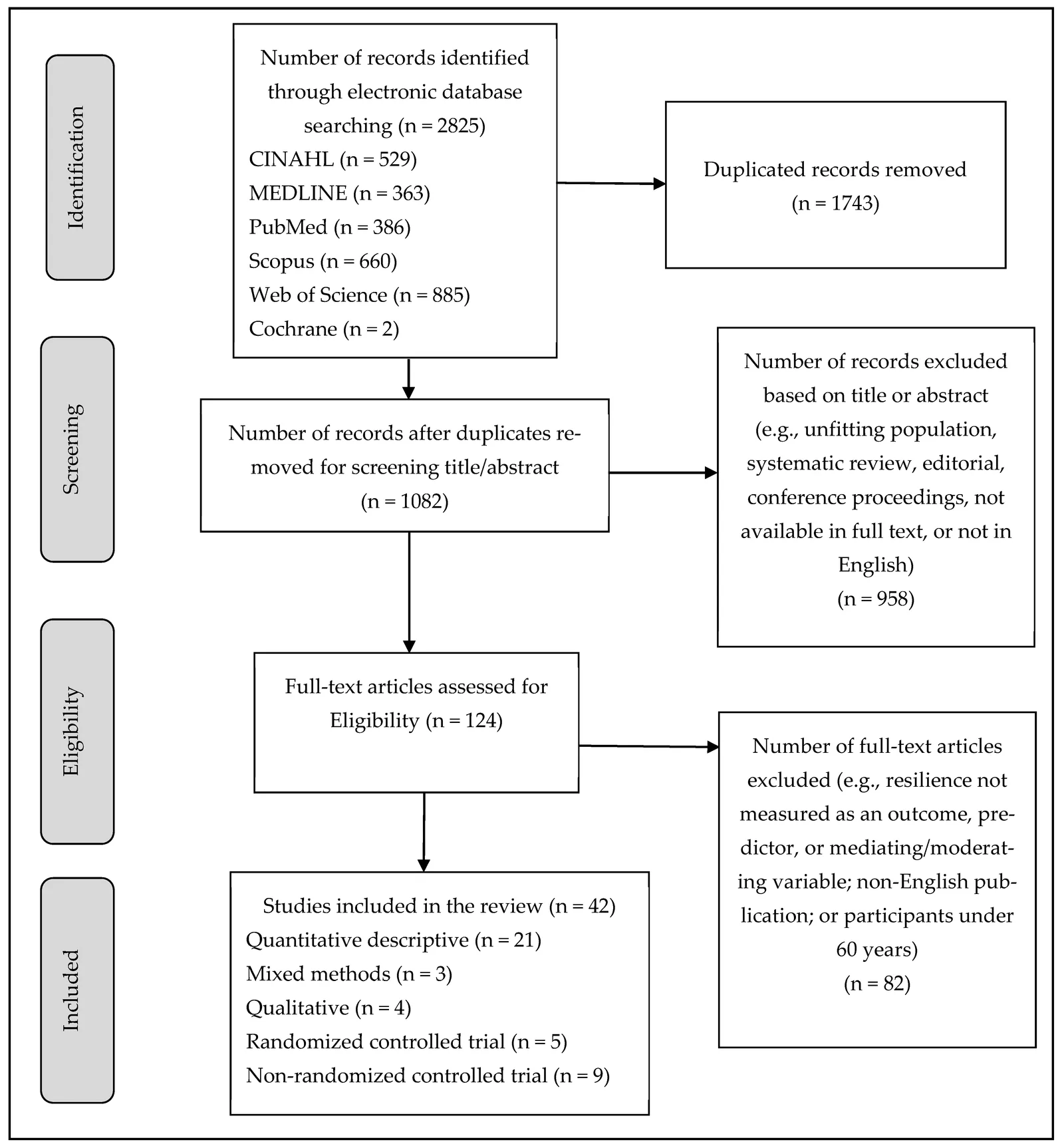
PRISMA flow diagram of the literature search.

The term “hardiness” was included to capture earlier resilience literature in which this construct was used as a conceptual precursor to resilience. A 10-year timeframe (January 2016–December 2025) was selected to capture contemporary understandings of resilience, reflecting recent theoretical developments and relevance to current ageing populations. Inclusion criteria required papers to have: (i) resilience measured using a named, validated construct or instrument, whether treated as an outcome, predictor, or mediating/moderating variable, in adults aged 60 years and above only, as the entire study sample; (ii) publication as English-language peer-reviewed empirical research in psychology, nursing, or social science. This strict age criterion was chosen to ensure that findings reflect older adults specifically, rather than being diluted by younger participants in mixed-age samples. However, this choice may have excluded mixed-age studies with informative subgroup analyses of older adults. As a result, some nuanced findings from larger mixed-age studies may not be captured in this review. This trade-off is considered further in Section 4.5. Exclusion criteria were: (i) editorials, commentaries, reviews, or theoretical papers; (ii) studies including participants under 60 years of age; and (iii) publications not available in English. Two independent reviewers screened titles and abstracts; disagreements were resolved via a third reviewer. The review was reported following the PRISMA checklist and flow diagram [24], presented in Figure 1.

### 2.2. Data Extraction

A standardised data extraction form captured study characteristics (author, year, country, design, setting), participant demographics, resilience definitions and instruments, predictor variables across psychological, social, health, and contextual domains, key findings, and intervention descriptions. Extraction was conducted independently by two reviewers, with discrepancies resolved through discussion or third-reviewer consultation.

### 2.3. Quality Assessment

Methodological quality was assessed using the Mixed Methods Appraisal Tool (MMAT), 2018 version [22]. Two reviewers rated each study independently against five criteria specific to its design (e.g., qualitative, quantitative, mixed methods), with each criterion scored as ‘Yes,’ ‘No,’ or ‘Cannot tell.’ Discrepancies were resolved through discussion, with a third reviewer consulted where consensus could not be reached, consistent with the disagreement-resolution procedure used for screening and data extraction. An overall score out of 5 was calculated based on the number of criteria met. We note that this overall score is a summary measure. It may mask specific domain weaknesses, such as limited researcher reflexivity in qualitative studies or selection bias in non-randomised studies. Summary MMAT scores for each study are reported in the Results section (Tables 3 and 4); item-level criterion ratings for each study are provided in Table S1.

The Integrative Review Framework assessed study relevance, methodological appropriateness, design rigour, clarity of findings, and generalizability [21]. Quality assessments informed interpretation and synthesis rather than serving as exclusion criteria, with attention to evidence strength and limitations [25]. Individual MMAT ratings for each included study are reported in the Results section to enable readers to evaluate the quality of the evidence base underpinning each finding. In response to peer review, the following Supplementary Materials have been prepared: complete per-database search strategies and dates of execution (Supplementary Section S1); the completed PRISMA 2020 checklist (Supplementary Section S2) [26]; the full data extraction table for all included studies (Supplementary Section S3) [27]; and a detailed description of the thematic coding framework with illustrative examples (Supplementary Section S4).

### 2.4. Data Synthesis

Two reviewers coded the included studies independently, using constant comparison. Codes were grouped into categories, and categories were grouped into the five themes shown in Table 1. Disagreements in coding were resolved through discussion, with a third reviewer consulted when needed. No formal weighting by quality score was applied during synthesis. Instead, quality ratings were used narratively to add context when interpreting findings of differing strength. We did not assess thematic saturation, as this concept is more commonly used in primary qualitative research than in evidence synthesis. Quantitative findings were summarised descriptively. Qualitative findings were grouped by population subgroup, including gender, age, geography, health status, and culture.

Representative studies illustrating each theme are presented in Table 2 to support transparency and allow readers to trace how individual studies informed the thematic categories in Table 1.

## 3. Results

### 3.1. Study Selection

The systematic search across six databases identified 2825 records in total (CINAHL *n* = 529; MEDLINE *n* = 363; PubMed *n* = 386; Scopus *n* = 660; Web of Science *n* = 885; Cochrane CENTRAL *n* = 2). After removing 1743 duplicates, 1082 unique records were screened by title and abstract, of which 958 were excluded, leaving 124 for full-text assessment. Of these, 82 were excluded for resilience not being measured as an outcome, predictor, or mediating/moderating variable, non-English publication, or participants under 60 years. The final review included 42 empirical studies. The PRISMA flow diagram (Figure 1) illustrates the complete selection process.

Although seven intervention studies were identified, this number reflects the stringency of the inclusion criteria rather than a lack of intervention research in this field. Many studies involving interventions in older adult populations either measured resilience as a secondary outcome or included participants under 60 years of age as part of mixed-age samples, rendering them ineligible. The search strategy was also expanded to include the term ‘hardiness,’ yet the number of eligible intervention studies remained consistent. The five themes identified through thematic analysis correspond directly to the three review objectives: Theme 1 addresses how resilience is conceptualised and measured (Objective 1); Themes 2 to 4 address individual, social, and contextual factors associated with resilience (Objective 2); and Theme 5 addresses interventions designed to promote resilience in older adults (Objective 3).

### 3.2. Study Assessment

As described in the Methods, quality was assessed using the MMAT [22]. Quality scores across the 42 included studies ranged from 2 to 5 out of 5, with approximately half of the studies achieving high ratings of 4 or 5. The lowest rating (2/5) reflects a single-group, pre-post quasi-experimental intervention study without a control condition [42]. Quantitative cross-sectional and longitudinal studies were generally rated at 4 or 5, reflecting adequate sampling, appropriate use of validated resilience measures, and clear reporting of findings. Qualitative and mixed-methods studies scored between 4 and 5, with some limitations noted in the areas of researcher reflexivity and transferability of findings. Quality ratings were used to inform the interpretation and weighting of evidence rather than as exclusion criteria, consistent with the integrative review approach. Individual MMAT ratings for each study are reported in Table 3 and Table 4. Table 3 presents the 35 studies addressing how resilience is conceptualised, measured and what individual, social, and contextual factors are associated with it. Table 4 presents the seven intervention studies, including intervention type, duration, resilience outcome measures, key findings, and quality ratings. Table 3 and Table 4 provide an overview of all 42 studies included in this review.

### 3.3. Study Characteristics

The 42 included studies were published between 2016 and 2025, spanning diverse older adult populations across multiple world regions. Geographically, Asia contributed the largest proportion of studies (*n* = 19), including research from China, Thailand, Japan, South Korea, Taiwan, Hong Kong, and Iran. North America contributed ten studies from the United States and Canada, Europe seven studies from Spain, Italy, Iceland, the United Kingdom, Sweden, Finland, and Turkey, South America five studies predominantly from Brazil, and one study from Egypt, together spanning 18 countries.

Study designs were methodologically diverse. Quantitative studies formed the largest group (*n* = 21), followed by qualitative studies (*n* = 4), mixed-methods studies (*n* = 3), randomised controlled trials (*n* = 5), and non-randomised quantitative studies (*n* = 9). Of these nine non-randomised studies, two were quasi-experimental interventions [42,43], whilst the remaining seven were observational longitudinal or cohort studies with no intervention component. Sample sizes varied considerably, ranging from 10 participants in a qualitative sub-sample to 77,395 in a large population-based survey, with most quantitative studies recruiting between 150 and 1300 participants.

**Table 3:** Characteristics of Studies Addressing Conceptualisation, Measurement, and Factors Associated with Resilience in Older Adults (*n* = 35).

No	Author (Year)	Country	Study Design	Sample (*n*) & Age	Resilience Measure(s)	Key Findings	Effect Size	MMAT
1	Achepohl et al. (2022) [33]	USA (California)	Mixed methods, sequential explanatory	Survey: *n* = 61 (35 online, 26 paper); Qual subsample: *n* = 12 interviews (6 low-resilience, 7 high-resilience, extreme-case sampling)	10-item Connor–Davidson Resilience Scale (CD-RISC), Posttraumatic Growth Inventory-Short Form	Participants categorized as having high resilience had long-held contemplative practice behaviors (meditation, prayer, gratitude) that helped them effectively adapt to the COVID-19 pandemic	N/A (extreme-case qualitative comparison; no formal effect size reported)	4/5 (Mixed methods)
2	Akatsuka & Tadaka (2021) [28]	Japan (Yokohama)	Quantitative, cross-sectional (scale development)	*n* = 1283 valid responses (of 3000 sampled), community-dwelling oldest-old ≥ 80 y	Newly developed Resilience Scale for Oldest-Old age (RSO, 9 items, 1 factor)	CFA identified a 9-item, 1-factor RSO with good fit; RSO positively correlated with PGC Morale Scale and self-anchoring scale for feeling life is worth living (SAS-WL)	Cronbach’s α = 0.800; GFI = 0.979, AGFI = 0.963, CFI = 0.973, RMSEA = 0.049; r = 0.492 (PGC, *p* < 0.001), r = 0.559 (SAS-WL, *p* < 0.001)	5/5 (Quantitative descriptive)
3	Cai et al. (2021) [44]	China (Shanghai)	Quantitative, cross-sectional	*n* = 196 disabled elders in nursing houses (102 male, 94 female), mean age 74.5 ± 6.81	10-item Connor–Davidson Resilience Scale (CD-RISC)	High resilience significantly enhanced sleep quality in disabled Chinese elders; perceived stress mediated the impact of resilience on sleep quality (2-step mediation model)	Mediation analysis significant (specific coefficients not extracted)	4/5 (Quantitative descriptive)
4	Chan et al. (2022) [45]	Hong Kong SAR, China	Mixed methods (quantitative survey + qualitative interviews); reclassified as Qualitative	Quant: *n* = 1067 (telephone survey, all adults); Qual: *n* = 10 older adults interviewed	No single named resilience scale; subjective well-being, COVID-19 worry, and social capital/interaction measures used	Older adults were less worried about COVID-19 infection and economic impact than younger adults, with better subjective well-being; 5 qualitative themes emerged, including life philosophy and positive coping strategies, suggesting stronger resilience in older adults during the pandemic	Not specified	4/5 (Qualitative, reclassified)
5	Hassani et al. (2017) [30]	Iran	Qualitative, descriptive phenomenology (Colaizzi’s method)	*n* = 22 hospitalized elderly with chronic disease; 24 interview sessions	N/A (qualitative)	4 themes: meaning of resilience in participants’ experiences; growth context as prologue to resilience; external factors contributing to resilience; personal factors to overcome illness	N/A (qualitative)	5/5 (Qualitative)
6	Hu et al. (2022) [46]	Taiwan	Quantitative, scale development/validation, cross-sectional	*n* = 200 older adults (mean age 76.4 ± 6.6 years; 51.0% women)	Newly developed Physical Resilience Instrument for Older Adults (PRIFOR; 16 items, 3 factors)	16-item PRIFOR showed satisfactory item fit, unidimensionality (Rasch model), and promising known-group and criterion-related validity	Cronbach’s α = 0.94	4/5 (Quantitative descriptive)
7	Kim et al. (2024) [47]	South Korea	Non-randomised, prospective longitudinal cohort (Korean Frailty and Aging Cohort Study)	*n* = 1826 (of 3014 total KFACS cohort), mean age 77.6 ± 3.7 years	Brief Resilience Scale (BRS)	Higher baseline BRS score associated with a lesser 2-year decline in global cognitive function (MMSE); no significant association with other specific cognitive domain measures	B = 0.175, 95% CI 0.025–0.325	5/5 (Non-randomised)
8	Laird et al. (2019) [48]	USA	Quantitative, cross-sectional (baseline data from 3 clinical trials)	*n* = 337 adults ≥ 60 y with major depressive disorder	Connor–Davidson Resilience Scale (CD-RISC)	EFA revealed a 4-factor model (grit, active coping self-efficacy, accommodative coping self-efficacy, spirituality); resilience associated with older age, lower cognitive functioning, greater cerebrovascular risk/medical comorbidity, higher QOL, minority race, and lower depression/apathy/anxiety	Correlations and general linear models (specific r/β not extracted)	3/5 (Quantitative descriptive)
9	Li & Ow (2022) [29]	Taiwan	Quantitative, scale development, cross-sectional	Main survey: *n* = 368 older adults; test–retest subsample: *n* = 76	Newly developed Resilience Scale for Older Adults (RSOA; 15 items, 4 constructs: personal strength, meaning/purpose of life, family support, social support)	RSOA showed good reliability and validity; all 4 constructs significantly correlated with life satisfaction of older adults	Cronbach’s α = 0.882	4/5 (Quantitative descriptive)
10	Li et al. (2025) [36]	China (Jinan, Shandong)	Quantitative, cross-sectional (moderated mediation)	*n* = 1758 older adults aged 60+	10-item Connor–Davidson Resilience Scale (CD-RISC-10)	Social support had a significant negative predictive effect on cognitive frailty; psychological resilience partially mediated the relationship between social support and cognitive frailty; education level moderated the social support–resilience relationship	B = −0.066 (*p* < 0.01, direct); B = −0.103 (*p* < 0.001, mediation); B = −0.184 to −0.315 across education levels (*p* < 0.05 to *p* < 0.01)	4/5 (Quantitative descriptive)
11	McKibbin et al. (2016) [34]	USA (rural/frontier Wyoming)	Quantitative, cross-sectional (mailed survey, random sample)	Random sample of registered voters aged 60+ in a frontier county	Resilience Scale (Wagnild-type, implied)	Family networks and mental health status significantly predicted resilience; mental health status moderated the relationship of family and friend networks with resilience—smaller networks linked to greater resilience only when mental health status was low	Family networks *p* = 0.024; mental health status *p* < 0.001; moderation: family *p* = 0.004, friend *p* = 0.021	3/5 (Quantitative descriptive)
12	Musich et al. (2022) [49]	USA	Quantitative, cross-sectional survey	*n* = 3573 adults aged 65+	Resilience level (low/medium/high) plus other protective factors index (purpose-in-life, optimism, locus of control, social connections)	Prevalence of low/medium/high resilience was 27%/29%/44%; strongest predictors of medium/high resilience were more protective factors, lower stress, and no depression; medium/high resilience associated with higher QOL and lower healthcare utilization/expenditures	Not extracted (regression-based associations reported in full text)	4/5 (Quantitative descriptive)
13	Özkan & Akça (2022) [31]	Turkey (Malatya)	Quantitative, cross-sectional (relational screening model)	*n* = 200 elderly (78 female, 122 male) residing at an Elderly Care Center	Brief Psychological Resilience Scale (BPRS), General Self-Efficacy Scale, Gratitude Scale	Positive relationship between self-efficacy, gratitude, and psychological resilience; gratitude had a mediating role in the relationship between self-efficacy and psychological resilience	Baron & Kenny mediation analysis with *t*-test (specific coefficients not extracted)	3/5 (Quantitative descriptive)
14	Palanbek Yavaş & Baysan (2025) [50]	Turkey	Quantitative, methodological (cross-cultural scale validation), cross-sectional	*n* = 566 individuals aged 65+	Turkish-adapted Resilience Scale for Older Adults (RSOA; 33 items, 4 subdimensions: Intrapersonal, Interpersonal, Spiritual, Experiential)	Turkish RSOA retained the original 4-factor structure; strong convergent validity with Quality of Life in Older Adults Scale; good internal consistency and split-half reliability	Cronbach’s α = 0.93; Guttman split-half = 0.723; r = 0.657 with QOL (*p* < 0.001)	4/5 (Quantitative descriptive)
15	Perez-Rojo et al. (2022) [51]	Spain	Quantitative, cross-sectional descriptive survey	*n* = 889 adults aged 60+	Resilience measured using a multidimensional framework (specific instrument not detailed in the original study) [49]	Older age, female sex, better perceived health, and not losing a loved one to COVID-19 associated with greater resilience; higher gratitude, personal growth, and life purpose, and lower depression, associated with greater resilience	Not extracted (correlational/regression analyses reported in full text)	4/5 (Quantitative descriptive)
16	Qiu et al. (2023) [52]	China (rural Anhui)	Quantitative, longitudinal, multilevel analysis (non-randomised)	*n* = 884 rural older adults aged 60+	Resilience operationalized as residuals of life satisfaction (Type-1) and depressive symptoms (Type-2) regressed on adversity, not a standard scale	43% exhibited both Type-1 and Type-2 resilience; self-rated health, financial satisfaction, and prestigious social networks were common predictors of both types; predictors varied by level of adversity (low/middle/high tertiles)	Multilevel regression coefficients (not extracted)	4/5 (Non-randomised)
17	Reis & Menezes (2017) [53]	Brazil (Jequié, Bahia)	Qualitative, phenomenological (Heideggerian)	*n* = 14 long-living older adults	N/A (qualitative)	Religiosity and spirituality (Bible reading, praying the rosary, prayer) served as resilience strategies for coping with adversity, health recovery/maintenance, personal/family protection, and satisfactory aging	N/A (qualitative)	5/5 (Qualitative)
18	Rodrigues & Tavares (2021) [54]	Brazil (Uberaba)	Quantitative, cross-sectional household survey	*n* = 808 elderly, majority female, aged 60–79	Resilience Scale (Wagnild & Young, Brazilian validated version for adults/elderly)	Total resilience score 78.06 (men 81.53; women 76.32); resilience associated with male sex, positive self-perceived health, greater participation in Advanced Activities of Daily Living (AADLs), fewer morbidities, and absence of depressive symptoms	Student’s *t*-test and multiple linear regression, *p* < 0.05 (specific β not extracted)	5/5 (Quantitative descriptive)
19	Sharaf et al. (2019) [55]	Egypt (Alexandria)	Quantitative, cross-sectional	*n* = 150 community-dwelling older adults	Resilience scale (specific named instrument not confirmed in extracted excerpt) & Ryff Psychological Well-being Scale	Psychological well-being strongly correlated with resilience; resilience significantly associated with 5 of 6 PWB dimensions (mastery, self-acceptance, personal growth, purpose in life, autonomy) and weakly with positive relationships	r = 0.70 (PWB–resilience); r = 0.54 (mastery); r = 0.53 (self-acceptance); r = 0.49 (personal growth & purpose); r = 0.36 (autonomy); r = 0.29 (positive relationships), all *p* < 0.001; β = 0.59 (adjusted PWB)	3/5 (Quantitative descriptive)
20	Shi et al. (2025) [56]	China (Chenzhou, Hunan)	Quantitative, cross-sectional (stratified cluster sampling)	*n* = 266 empty-nest older adults (urban, suburban, rural communities)	Connor–Davidson Resilience Scale (CD-RISC)	Healthy ageing score 134.34 ± 20.06 (of 175); resilience score 72.52 ± 13.97 (of 100); living arrangement, income source, and frequency of social activities significantly associated with healthy ageing; adding resilience to the model increased explanatory power by 29.8%	F = 25.454, *p* < 0.001	4/5 (Quantitative descriptive)
21	Sigurðardóttir et al. (2022) [37]	Iceland (Northern)	Quantitative, cross-sectional, population-based	*n* = 180 (105 urban, 75 rural), aged ≥65	Connor–Davidson Resilience Scale (CD-RISC)	Urban dwellers had more education, better access to organized physical/social activities, and higher resilience than rural dwellers; better health literacy and better mental health increased resilience	Urban vs. rural resilience *p* < 0.001; education *p* < 0.001; physical/social activity access *p* < 0.001; multivariate health literacy & mental health effect *p* < 0.001	5/5 (Quantitative descriptive)
22	Silva Júnior et al. (2019) [57]	Brazil (Campina Grande)	Quantitative, cross-sectional	*n* = 86 urban elderly	Resilience Scale (Wagnild & Young) & Social Support Scale	High resilience (M = 134.37, SD = 16.6) and moderate social support (M = 17.36, SD = 2.77); resilience significantly associated only with religion; weak positive correlation between the independence/determination factor and social support; social support was not a predictive variable for resilience in regression	χ^2^ = 0.30, *p* = 0.027 (resilience–religion); correlation *p* = 0.005 (independence/determination–social support)	3/5 (Quantitative descriptive)
23	Silveira et al. (2018) [35]	Brazil (Belo Horizonte)	Qualitative (“signs, meanings and actions” model)	*n* = 13 robust/pre-frail elderly, aged 69–86	N/A (qualitative)	Resilience constructed through: (a) construction of bonds with family; (b) reinvention of the self after trauma; (c) religiosity (Catholic, Evangelical, Spiritualist) as a source of strength and belonging	N/A (qualitative)	5/5 (Qualitative)
24	Springfield et al. (2022) [58]	USA	Quantitative, cross-sectional (secondary analysis)	*n* = 77,395 women aged 62+ (Women’s Health Initiative Extension Study)	Brief Resilience Scale (short version)	Higher resilience associated with younger age, fewer stressful life events, more resources; Black/African American women reported highest resilience; most important resilience-related resources were psychological (control beliefs, energy, personal growth, mild/no forgetfulness, sense of purpose); race/ethnicity moderated the resilience–energy relationship	Overall interaction *p* = 0.0017 (race/ethnicity × energy); elastic net regression (coefficients not extracted)	3/5 (Quantitative descriptive)
25	Thongkhum et al. (2022) [59]	Thailand	Mixed methods (sequential exploratory: qualitative FGD → quantitative survey)	Qual: *n* = 6 elderly (60–69 y) with chronic disease & depression. Quant: *n* = 310 elderly with chronic disease & depression	Newly developed Resilience Scale for Thai Elderly with Chronic Disease & Depression (21 items)	Resilience defined by 3 themes (My Characteristics, My Abilities, My Dependencies) composed of 9 categories; new scale showed good item-total correlations and construct validity (CFA fit the data well)	Cronbach’s α = 0.85; CFA: χ^2^ = 161.51, df = 186, *p* = 0.90, RMSEA = 0.000	4/5 (Mixed methods)
26	Upasen et al. (2024) [60]	Thailand	Quantitative, cross-sectional (structural equation/path analysis)	*n* = 964 older adults (multi-stage random sampling)	Thai Elderly Resilience Scale, Social Support Scale, Thai Geriatric Mental Health Assessment	Older adults had high levels of resilience, social support, and mental health; social support and resilience directly and significantly influenced mental health; social support also had a significant indirect effect on mental health via resilience	Path analysis coefficients (not extracted)	4/5 (Quantitative descriptive)
27	Viglund et al. (2021) [61]	Finland & Sweden	Longitudinal, quantitative (2 waves; non-randomised)	*n* = 4023, born 1930/1935/1940/1945	Inner Strength Scale (ISS)	Crises/diseases positively predicted inner strength over time and negatively predicted well-being; inner strength and well-being had a reciprocal positive relationship; health/function positively predicted inner strength	Cross-lagged panel SEM path coefficients (not extracted)	4/5 (Non-randomised)
28	Vlasova et al. (2018) [32]	USA	Quantitative, cross-sectional (neuroimaging)	*n* = 70 older adults with major depressive disorder	Connor–Davidson Resilience Scale (CD-RISC), 4-factor EFA solution: grit, active coping self-efficacy, accommodative coping self-efficacy, spirituality	The “grit” resilience factor was positively associated with white matter integrity (fractional anisotropy) in the genu of the corpus callosum and cingulum; no white matter integrity correlates were found for the other 3 resilience factors	F(1,64) = 7.54, *p* = 0.008 (genu corpus callosum–Grit); F(1,64) = 4.47, *p* = 0.04 (cingulum–Grit)	3/5 (Quantitative descriptive)
29	Wang et al. (2024) [62]	China	Non-randomised, prospective cohort (Chinese Longitudinal Healthy Longevity Survey)	*n* = 4935 participants aged 65+	5-item psychological resilience measure (CLHLS)	Higher psychological resilience independently associated with lower all-cause mortality and cause-specific mortality (cardiovascular, respiratory, and other causes, but not cancer); dose–response relationship; effect more pronounced among economically independent older adults	HR = 0.74 all-cause (95% CI 0.67–0.82); HR = 0.74 CVD; HR = 0.63 respiratory; HR = 0.69 other causes	5/5 (Non-randomised)
30	Windle et al. (2020) [63]	Wales, UK	Mixed methods, two-stage cross-sectional (CFAS Wales cohort)	*n* = 20 semi-structured interviews (subsample of quantitatively defined resilient participants aged 65+, identified from larger cohort with high well-being despite co-morbidity)	Resilient status defined quantitatively via well-being relative to co-morbidity (ecopsychosocial framework); qualitative interviews not scale-based	4 life-experience themes: early years as formative; work/employment as formative; adverse events and experiences; caring experiences. Four resilience mechanisms: character & self-identity; approach to life & insight; meaningful relationships & belonging	N/A (qualitative)	4/5 (Mixed methods)
31	Wister et al. (2022) [64]	Canada	Quantitative, longitudinal (non-randomised); Canadian Longitudinal Study on Aging (CLSA)	*n* = 9211 older adults with 2+ chronic conditions (multimorbidity)	Multimorbidity resilience index (functional, social, and psychological sub-indices)	Multimorbidity resilience index inversely associated with pandemic comprehensive impact and personal worry; psychological resilience sub-index most consistently and inversely associated with impact; social resilience sub-index weakly positively associated with worry; functional resilience not associated with either outcome	OR = 0.83–0.89 across models (*p* < 0.001 or *p* < 0.05)	4/5 (Non-randomised)
32	Wister et al. (2024) [38]	Canada	Quantitative, longitudinal (Canadian Longitudinal Study on Aging)	*n* = 13,064 older adults with multimorbidity, aged 65+	Connor–Davidson Resilience Scale (CD-RISC)	Strongest resilience associations: higher self-rated health, greater sleep satisfaction, better appetite, higher household income, more relatives/friends, being overweight, fewer housing problems, fewer skipped meals; weaker associations for non-smoking, less alcohol, less pain, non-married status, and being Canadian-born	Linear regression coefficients (not extracted)	4/5 (Quantitative descriptive)
33	Wosiack et al. (2018) [65]	Brazil (Ivoti)	Quantitative, cross-sectional	*n* = 161 elderly, both sexes	Resilience Scale (Wagnild & Young)	Resilience correlated with quality of life, basic psychological needs, wisdom, and cognition, and inversely with depression; regression showed resilience increase associated with personal competence, autonomy, wisdom, cognitive performance, and life activities	Spearman correlations & linear regression (specific coefficients not extracted)	3/5 (Quantitative descriptive)
34	Ye et al. (2024) [66]	China (Shanghai)	Non-randomised, prospective cohort study	*n* = 4033 community-dwelling older adults, mean age 71.0 ± 6.1	25-item Connor–Davidson Resilience Scale (CD-RISC)	Higher psychological resilience (PR) associated with reduced frailty progression, particularly among those with higher baseline frailty; changes in PR (ΔPR) were nonlinearly associated with both frailty improvement and deterioration	β = −0.136 (95% CI −0.214 to −0.057, high baseline frailty); OR = 1.28 (improvement, 95% CI 1.05–1.57); OR = 1.30 (ΔPR improvement); OR = 0.74 (ΔPR deterioration)	5/5 (Non-randomised)
35	Carta et al. (2022) [67]	Italy (Cagliari, Sardinia)	Non-randomised (post-RCT follow-up cohort and nested case–control study)	*n* = 79 (of 105 RCT completers) entered the COVID-19 lockdown follow-up phase; mean age 72.3 ± 4.7 (at week 12), 56% women	Brief Symptom Rating Scale (BSRS) for Social and Behavioral Rhythms (SBRs) functioning; Patient Health Questionnaire-9 (PHQ-9) for depression as inverse resilience marker	Frequency of major depressive episode (MDE) did not significantly change before vs. during lockdown; those with impaired SBR functioning (BSRS >1 SD) at week 12 had an inflated risk of depressive episode during lockdown; overall, older adults were resilient during the initial pandemic phase while pre-lockdown functioning was preserved, but impairment of daily rhythms was associated with reduced resilience later	MDE frequency: OR = 2.60, 95% CI 0.87–9.13; impaired SBR → depressive episode: OR = 5.6, 95% CI 1.5–21.4	3/5 (Non-randomised)

Notes: CD-RISC = Connor–Davidson Resilience Scale; RSO = Resilience Scale for Oldest-Old Age; RSOA = Resilience Scale for Older Adults; PRIFOR = Physical Resilience Instrument for Older Adults; BPRS = Brief Psychological Resilience Scale; BRS = Brief Resilience Scale; ISS = Inner Strength Scale; MMAT = Mixed Methods Appraisal Tool; AADL = Advanced Activities of Daily Living.

**Table 4:** Characteristics of Studies Examining the Efficacy of Resilience-Promoting Interventions in Older Adults (*n* = 7).

No.	Author (Year)	Country	Study Design	Sample (*n*) & Age	Intervention Type	Sessions & Duration	Follow-Up Period	Resilience Measure(s)	Key Findings	Effect Size	MMAT
1	Han & Koo (2018) [42]	South Korea	Quasi-experimental (single group, pre-post; no control group)	*n* = 20; mean age 71.45; 65–75 years; 75% female	Nature-based forest healing programme combining walking and meditation in an urban forest setting	4 sessions; 90 min each; twice weekly	2 weeks total; no follow-up reported	KRQ-53 (53-item; 5-point Likert)	Resilience improved significantly post-programme; greatest gains in self-regulation and interpersonal ability	Pre M = 168.4 vs. post M = 178.0; t = −4.554, *p* < 0.05; self-regulation: t = −2.630, *p* = 0.016; interpersonal ability: t = −4.080, *p* = 0.001	2/5
2	Kukihara et al. (2020) [39]	Japan	RCT (three-arm: exercise, mindfulness-yoga, control)	*n* = 55; mean age ≈70; 65+ years	Structured exercise vs. mindfulness-based yoga vs. control (TV watching)	Weekly sessions; 50 min each	12 weeks; post-intervention only, no extended follow-up	Wagnild & Young RS (25-item; 7-point Likert; α = 0.96)	Both exercise and yoga significantly improved resilience over control; resilience fully mediate the reduction in minor psychiatric disorders in both active arms	F(2,54) = 5.03, *p* = 0.01; exercise M = 64.42 vs. control M = 48.77; yoga M = 64.20 vs. control M = 48.77; Sobel z = 2.41, *p* = 0.008	4/5
3	Taherkhani et al. (2023) [68]	Iran	RCT (parallel group: intervention vs. control)	*n* = 100 (intervention *n* = 50; control *n* = 50); 60–70 years	Positive thinking training in group sessions; cognitive reframing and optimistic thinking skills	8 sessions; 90 min each; once weekly	8 weeks; baseline, 1-week post, and 2-month follow-up	CD-RISC (25-item; 5-point Likert; α = 0.97)	Resilience and life satisfaction significantly higher in intervention group at post-test and 2-month follow-up, demonstrating durable gains	Cohen’s d = 2.16 (post-test); d = 1.15 (2-month follow-up); η^2^ = 0.50	4/5
4	Treichler et al. (2020) [43]	USA	Pragmatic modified stepped-wedge trial	*n* = 89; mean age 84.9 (SD = 7.0); senior housing residents	‘Raise Your Resilience’ (RYR)—group ntervention incorporating savoring, gratitude, and value-based activities	3 sessions; 90 min each	1 month active; 1-month control period; 3-month post-intervention follow-up	CD-RISC (10-item); PSS; SF-12; SD-WISE; LOT-R	Resilience and perceived stress improved significantly at 3-month follow-up; wisdom subscales also improved; no significant changes in well-being, happiness, or optimism	CD-RISC Cohen’s d = 0.115; PSS Cohen’s d = −0.192; SD-WISE Cohen’s d = 0.251	3/5
5	Wu et al. (2024) [69]	Taiwan	Single-blind, parallel-group RCT	*n* = 63 (intervention *n* = 31; control *n* = 32); mean age 72.11; 65–92 years	Web-based resilience-enhancing programme (Resilience in Illness Model) via LINE platform; psychoeducation, peer mentorship, skill-building	4 sessions; 60 min each; once weekly	4 weeks; baseline (T0), 4 weeks (T1), and 12 weeks (T2)	CRS (25-item; 7-point Likert); PASE; WBS	No significant between-group differences in resilience, hysical activity, or well-being at any time point; programme was feasible and acceptable but did not demonstrate efficacy	No meaningful effect sizes; all between-group differences non-significant	3/5
6	Lavretsky et al. (2022) [40]	USA	Randomized controlled trial (single-blind)	*n* = 178 randomized (89 Tai Chi Chih, 89 Health Education/Wellness); 125 completed 3-mo, 117 completed 6-mo	Tai Chi Chih (TCC) vs. Health Education and Wellness training (HEW), both combined with stable standard antidepressant treatment	Weekly sessions, 60 min each	12 weeks active; naturalistic follow-up to 6 months	Connor–Davidson Resilience Scale (CD-RISC)	Both TCC and HEW combined with antidepressants improved depression; CD-RISC scores improved significantly within both groups at 3 and 6 months, with no significant between-group difference; TCC showed greater improvement in general health than HEW	CD-RISC change: TCC +4.71 (SD 13.25) vs. HEW +3.66 (SD 9.75) at 3 mo, F(1174) = 0.3, *p* = 0.6; Cohen’s d = −0.09 (3 mo), −0.06 (6 mo)	4/5
7	Tsai et al. (2025) [41]	Taiwan	Mixed methods (RCT with embedded qualitative interviews)	*n* = 69 randomized (intervention/control) from a senior fitness club; intervention group completed ≥6 months of smart sports equipment course; *n* = 16 qualitative interview subsample	Intelligent/smart sports equipment (IoT, big data, AI-driven) exercise programme	Structured phase: 24 h over 12 weeks, embedded within overall engagement	6 months; baseline and 6-month assessment	Chinese version of the Resilience Scale (CRS; 6 items), translated from the Brief Resilience Scale (Smith et al.)	Resilience scores in the intervention group increased significantly from baseline to 6 months; qualitative themes: (1) transition from adapting to internalizing digital technology, (2) exercises aligned with participants’ needs, (3) fostering of close peer relationships	21.35 ± 4.37 (baseline) to 23.35 ± 5.4 (6 months), *p* = 0.023	4/5

Notes: RCT = Randomised Controlled Trial; CD-RISC = Connor–Davidson Resilience Scale; RS = Wagnild & Young Resilience Scale; KRQ-53 = Korean Resilience Quotient-53; CRS = Chinese Resilience Scale; PASE = Physical Activity Scale for the Elderly; WBS = Well-Being Scale; RYR = Raise Your Resilience; PSS = Perceived Stress Scale; SD-WISE = San Diego Wisdom Scale; LOT-R = Life Orientation Test Revised; MMAT = Mixed Methods Appraisal Tool; MMSE = Mini-Mental State Examination.

### 3.4. Conceptualisation and Measurement of Resilience

Across the 42 included studies, resilience was conceptualised in three broad ways: as a stable personal trait, a dynamic adaptive process, and a measurable outcome of successful coping with adversity. Trait-oriented studies emphasised enduring attributes such as self-efficacy, perseverance, and sense of purpose, often operationalised through the CD-RISC’s underlying factor structure of grit, coping self-efficacy, and spirituality [48], or through purpose-built trait inventories such as the RSO for the oldest-old [28]. Process-oriented studies instead understood resilience as developing through sustained engagement between the person, their relationships, and their broader environment, evident in accounts of reinventing oneself in older age [35] and in multidimensional frameworks linking functional, psychological, and social resilience sub-indices to fluctuating health circumstances [64]. A smaller number operationalised resilience as the maintenance or recovery of well-being in the face of chronic illness, bereavement, frailty, or population-level adversity, including stability of depressive symptoms and quality of life through the COVID-19 pandemic [67], life satisfaction thresholds despite multimorbidity [63], and residual well-being unexplained by adversity exposure [52].

More than twenty distinct instruments were identified, reflecting measurement heterogeneity. The CD-RISC and its abbreviated versions were most widely used, followed by the Wagnild and Young Resilience Scale and the Brief Resilience Scale. Several age-specific and culturally adapted tools were developed or validated, including the RSO for the oldest-old in Japan [28], the RSOA in Taiwan and Turkey [29,50], the PRIFOR for hospitalised older adults in Taiwan [46], and a Thai resilience scale for older adults with chronic illness and depression [59]. Qualitative studies further revealed dimensions beyond quantitative measurement, including patience, trust in God, reinvention of self, acceptance of chronic disease, and a philosophy of life that reframed hardship as an ordinary part of ageing [30,35,45,53,63]. Measurement also extended beyond self-report in at least one study: a neuroimaging secondary analysis found that the grit component of resilience was positively associated with white matter integrity in a prefrontal-connecting tract, suggesting that self-reported resilience factors may have identifiable biological correlates [32].

Longitudinal evidence supported process-oriented conceptualisations. A six-year panel study found that inner strength and well-being reciprocally reinforced each other, and that crises were associated with increases rather than declines in inner strength [61]. Changes in resilience were significantly associated with frailty trajectories over three years, with resilience declines more strongly predicting deterioration than improvements predicted recovery [67]. Higher baseline resilience was also associated with less cognitive decline over two years [47] and reduced all-cause mortality over 3.2 years [62], collectively supporting resilience as a dynamic process rather than a fixed trait.

### 3.5. Individual Psychological Factors Predicting Resilience

Individual psychological characteristics were among the most consistently identified correlates of resilience. Self-efficacy and self-esteem emerged as particularly prominent predictors. Perceived personal control predicted resilience in a cross-sectional study of 200 older adults in Turkey, with gratitude showing a statistically significant indirect effect in this relationship, though the cross-sectional design precludes confirmation of mediation [31]. Among 77,395 racially diverse older women in the United States, perceived control over beliefs, personal energy, and opportunities for personal growth were the most influential modifiable determinants of resilience [58].

Gratitude, spirituality, and meaning-making were consistently associated with higher resilience across diverse cultural contexts. High-resilience older adults in the United States drew upon contemplative practices including meditation and gratitude to sustain equanimity during COVID-19 [33]. Older adults in Iran described spirituality and trust in God as primary resilience sources in the face of chronic illness [30], whilst the oldest-old in Brazil articulated faith as providing spiritual co-presence that filled the existential void of accumulated losses [53]. In Spain, gratitude, personal growth, and life purpose were the strongest resilience predictors even under pandemic stress [51], and spirituality operated alongside acceptance and social support as a core coping resource among older adults with chronic illness in Thailand [59].

Sense of coherence was identified as a significant longitudinal predictor of resilience trajectories, with social support partly mediating this relationship over time, providing stronger evidence than cross-sectional associations alone for sense of coherence as a modifiable intervention target [70]. Among depressed older adults, four resilience factors: grit, active coping self-efficacy, accommodative coping self-efficacy, and spirituality, remained negatively associated with depression and apathy and positively associated with quality of life, suggesting that psychological resilience resources remain accessible even in clinical vulnerability [48]. Digital literacy represents an emerging individual-level resource; among community-dwelling older adults in China, digital skills were positively associated with self-efficacy, social inclusion, and autonomy, whilst limited technological competence was associated with social exclusion and reduced access to health information [71].

### 3.6. Social and Relational Factors Supporting Resilience

Family relationships and social networks were among the most consistently identified protective factors, though the nature and strength of these associations varied by cultural context and setting. In rural United States, family networks and mental health status were the strongest resilience predictors, with family support buffering the adverse effects of poor mental health [34]. Perceived relationship quality consistently showed stronger associations with resilience than quantity alone. Among the oldest-old in Brazil, grandchild relationships provided essential meaning during frailty, with resilience described as a fabric woven from family bonds, religious experience, and personal reinvention [35].

Social support operated not only as a direct protective factor but also through strengthening broader psychological resources. A longitudinal study found that perceived social support partly mediated the relationship between sense of coherence and resilience over time [70]. Among 1758 older adults in China, social support had a significant negative predictive effect on cognitive frailty, an effect partially mediated by psychological resilience and moderated by education level, with the protective effect of social support on resilience strengthening at higher educational attainment [36]. In a large Canadian cohort (*n* = 13,064), social engagement and community participation partially offset the adverse effects of multimorbidity on resilience [38].

Spiritual and religious engagement functioned as a primary relational and meaning-making resource across multiple cultural contexts. Spirituality, patience, and hope were central to accepting chronic illness among older adults in Iran [30], whilst faith provided spiritual co-presence mitigating existential isolation among the oldest-old in Brazil [53]. In Thailand, religion and family support were the primary external resilience dependencies alongside internal characteristics such as patience and coping ability [59], and internal factors including determination and faith were more strongly predictive of resilience than objective social support levels alone [57].

Community participation further contributed to resilience across diverse settings. Urban older adults in Iceland demonstrated higher resilience than rural counterparts, attributable not to geographic location itself but to differential access to organised social and physical activities [37]. In Brazil, participation in advanced activities of daily living and community clubs was associated with higher resilience scores [54]. During COVID-19 in Hong Kong, stable pension income, a settled life philosophy, and telecommunications adoption collectively buffered pandemic stressors on resilience and life satisfaction [45]. Early life employment histories and meaningful relationships were also associated with older adults reframing illness as a manageable challenge rather than an insurmountable threat [63], suggesting that social foundations of resilience are laid across the life course.

### 3.7. Health Status, Education, and Contextual Characteristics

Health status and functional independence were among the most consistently identified contextual correlates of resilience. Higher resilience was significantly associated with living at home without long-term care needs among the oldest-old in Japan [28], and positive self-perceived health was one of the strongest resilience predictors during COVID-19 in Spain [51]. Among disabled older adults in nursing homes in China, higher resilience significantly enhanced sleep quality, with perceived stress acting as a partial mediator [44]. Longitudinal studies added important depth; declining resilience was more strongly associated with worsening frailty than improving resilience was with recovery [66], higher baseline resilience was independently associated with less cognitive decline over two years [47], and higher resilience was associated with reduced all-cause, cardiovascular, and respiratory mortality in a Chinese cohort of 4935 older adults [62], collectively suggesting resilience may function as an active protective mechanism influencing health trajectories in later life. Higher resilience was also linked to lower healthcare utilisation and expenditure, with each additional protective factor increasing the likelihood of high resilience among 3573 older adults in the United States [49].

Educational attainment, marital status, and economic resources constituted enabling contextual conditions for resilience. Financial security bolstered resilience during COVID-19 in Hong Kong [45], whilst multimorbidity clustering was associated with reduced resilience across all disease groupings in a large Canadian cohort, though social engagement partially moderated this association [65]. Among empty-nest older adults in China, living arrangement, pension income, and frequency of social activity were significant correlates of healthy ageing alongside resilience [56]. Gender differences emerged inconsistently; female gender was associated with higher resilience in Spain [51], whilst higher resilience was found among males in Brazil [54], suggesting gender shapes the pathways through which resilience is expressed rather than determining resilience capacity intrinsically.

Geographic context and digital access also shaped resilience. Urban older adults demonstrated higher resilience than rural counterparts in Iceland, attributable to differential access to organised social and physical activities rather than geographic location itself [37]. Among 77,395 racially diverse older women in the United States, African American women reported the highest resilience levels, with perceived control, personal energy, and opportunities for growth as the most influential modifiable determinants [58]. Social support, mental health, and resilience were highly interrelated among 964 older adults in Thailand, with resilience directly and indirectly shaping mental health outcomes [60]. Digital skills were positively associated with self-efficacy, social inclusion, and health information access, whilst limited technological competence was associated with social exclusion and reduced autonomy [71], suggesting that digital inequalities may increasingly constitute a form of contextual disadvantage with direct implications for resilience in ageing populations.

### 3.8. Interventions Promoting Resilience in Older Adults

Seven studies examined structured interventions designed to promote resilience in older adults, comprising five randomised controlled trials, one pragmatic trial, and one quasi-experimental study. Intervention types included nature-based programmes, physical exercise, mindfulness-based yoga, positive thinking training, Tai Chi, technology-supported exercise, and web-based multicomponent programmes. Whilst findings should be interpreted cautiously given modest samples and variable follow-up periods, the seven studies collectively offer preliminary but meaningful evidence for understanding what approaches may promote resilience. Nature-based and physical activity interventions showed consistent promise. A quasi-experimental forest healing programme combining walking and meditation over four sessions significantly improved resilience among 20 older adults in South Korea, with the greatest gains in self-regulation and interpersonal ability [42]. A Japanese RCT comparing structured exercise against mindfulness-based yoga over 12 weeks found that both interventions significantly improved resilience, and notably, resilience fully mediated the reduction in minor psychiatric disorders in both arms, establishing resilience as a functional mechanism rather than an incidental outcome [39]. An Iranian RCT evaluating an eight-week positive thinking training programme found significant and durable improvements in resilience and life satisfaction sustained at two-month follow-up, demonstrating that cognitive skills remain trainable in older age [68].

A pragmatic trial in the United States evaluating a manualized group intervention incorporating savoring, gratitude, and value-based activities found significant resilience improvements at three-month follow-up, though effect sizes were small [43]. A US RCT comparing Tai Chi Chih against health education and wellness training, both combined with standard antidepressant treatment, found significant within-group improvements in resilience at three and six months in both arms, with no significant between-group difference [40]. In Taiwan, a mixed-methods RCT of an intelligent, technology-supported exercise programme found significant resilience gains at six months, with qualitative interviews revealing themes of digital adaptation, needs-aligned exercise, and peer connection [41]. Importantly, one web-based RCT from Taiwan found no significant resilience improvements, with both groups showing similar trajectories across all time points [69], a reminder that digital delivery alone does not guarantee effectiveness and that programme content, dose, and participant characteristics remain critical moderators of intervention outcomes.

Across all seven studies, group-based delivery formats featured prominently and may have contributed to resilience gains through the simultaneous cultivation of social connectedness. Intervention durations ranged from four sessions to six months, and two studies [40,68] reported follow-up data extending to two and six months respectively, beyond the immediate post-intervention period. Sample sizes ranged from 20 to 178 participants, and none reported health economic data. Together, the available evidence offers preliminary support for physical activity, mindfulness-based approaches, technology-supported exercise, and psychological skill-building as promising modalities for resilience promotion, whilst underscoring the urgent need for larger, more rigorous trials with active control conditions, standardised outcome measures, sufficient follow-up periods, and health economic components.

### 3.9. Associations Between Resilience and Well-Being Correlates

Across the 42 included studies, resilience was consistently associated with a broad range of health and well-being outcomes, spanning psychological well-being, quality of life, life satisfaction, mental health, cognitive function, frailty, and mortality. Whilst most of these associations derive from cross-sectional studies and cannot establish causal direction, their convergence across diverse populations, cultural contexts, and methodological approaches lends them meaningful descriptive weight.

Quality of life and functional autonomy showed consistent positive associations with resilience across multiple countries and settings. Among community-dwelling older adults in Brazil, resilience was positively correlated with quality of life, functional autonomy, and cognitive performance [65], whilst higher resilience was associated with better self-reported quality of life and reduced apathy even among older adults with major depressive disorder in the United States [48]. Psychological well-being demonstrated particularly strong associations; autonomy, environmental mastery, and personal growth collectively accounted for 48% of resilience variance in Egypt, with inner psychological assets appearing more influential than external resources [55]. Mental health outcomes showed consistent inverse associations with resilience across clinical and community samples, with higher resilience associated with lower depression, reduced anxiety, and diminished psychological distress [48,72]. Higher resilience was also linked to lower healthcare utilisation, fewer emergency visits, and reduced medical expenditure among 3573 older adults in the United States [49]. In Hong Kong, resilience buffered the adverse effects of COVID-19 on life satisfaction, with stable life philosophy, economic security, and telecommunications adoption sustaining both resilience and well-being under prolonged adversity [45]. Among 77,395 racially diverse older women in the United States, African American women demonstrated particularly high resilience levels attributable to modifiable personal resources, underscoring the importance of culturally affirming and strengths-based approaches [58].

Longitudinal evidence more substantively positions resilience as an active contributor to health trajectories. A six-year panel study found that inner strength and well-being reciprocally reinforced each other, with crises associated with increases rather than decreases in inner strength [61]. Higher baseline resilience was associated with less cognitive decline over two years, with protective effects partially moderated by social engagement [47]. Declining resilience was more strongly associated with worsening frailty than improving resilience was with recovery, suggesting an asymmetric relationship in which resilience loss may carry greater physical health costs than resilience gain brings benefits [66]. A large Chinese cohort found that higher resilience was independently associated with reduced all-cause mortality, including cardiovascular and respiratory mortality, with a dose–response pattern suggesting each unit increase in resilience score corresponded to a measurable reduction in mortality risk [62]. These longitudinal findings collectively position resilience not merely as a correlate of good health but as a potentially protective mechanism influencing physical health trajectories, cognitive function, and survival in older adulthood.

These associations are consistent across diverse populations. Combined with early evidence that resilience can be modified, this supports further research into resilience promotion as a possible health priority for ageing populations. At this stage, this remains a research priority rather than a confirmed policy priority.

## 4. Discussion

### 4.1. Conceptual Diversity and Measurement Heterogeneity in Resilience Research

Resilience in older adults continues to be understood in different ways, and this review found three broad orientations across the included studies: resilience as a stable personal trait, a dynamic adaptive process, and a measurable outcome of successful coping with adversity. How resilience is conceptualised matters in practice; it shapes the tools used to measure it, the interventions designed to promote it, and how outcomes are interpreted in clinical and community settings [73]. Trait-oriented instruments such as the CD-RISC and the Wagnild and Young Resilience Scale are widely used. However, they treat resilience as a fixed personal characteristic. This differs from the qualitative findings in this review, which show that resilience changes in response to relationships, health, and life circumstances [73,74]. These trait-based scales were also developed mainly in healthy, community-dwelling samples. They may not fully capture resilience in older adults living with disability, dependency, or frailty. They may also miss culturally embedded forms of coping, such as faith-based practices or family duty, that do not map onto standard scale items. This is an important gap for geriatric assessment, where many older adults face exactly these conditions.

Among the new contributions of this review, six longitudinal studies collectively show that resilience is not static but shifts over time in response to adversity, health changes, and social circumstances. One six-year panel study of 4023 older adults in Finland and Sweden found that inner strength grew through engaged coping with adversity rather than diminishing [61], which challenges the common assumption that ageing is associated with resilience decline. A stress reactivity and recovery perspective was less commonly applied across the included studies, though findings from studies tracking frailty progression, cognitive decline, and bereavement sit reasonably well within this framework [75]. This points to a gap worth addressing; future measurement tools could usefully incorporate recovery-oriented indicators so that the full picture of how older adults respond to and recover from adversity is captured, not just their baseline levels of resilience.

One of the clearest challenges to emerge from this review is measurement heterogeneity: more than twenty different instruments were used across the 42 included studies, making it difficult to compare findings in any straightforward way, and this problem is not new [73,74]. Several research teams have developed or validated instruments specifically designed for older populations, including the RSO for adults aged 80 and above in Japan [28], the RSOA in Taiwan and Turkey [29,50], the PRIFOR for hospitalised older adults in Taiwan [46], and a Thai scale for older adults living with chronic illness and depression [59]. These efforts reflect a growing recognition that generic resilience tools may not capture what matters most to older people in different cultural settings, though how well these instruments travel across cultures still needs proper testing. Qualitative studies in this review revealed dimensions of resilience that standardised scales rarely reflect, such as patience, trust in God, spiritual co-presence, and the quiet reinvention of self in the face of loss [30,53]. This is a meaningful finding because it suggests that much of what sustains older adults through adversity remains invisible to quantitative measurement. Reaching an international consensus on what resilience means across cultures and developing tools that can capture it faithfully remains two of the most pressing tasks ahead for researchers in this field.

### 4.2. Factors Supporting Resilience: Psychological, Social, and Contextual

Understanding what predicts or correlates with resilience is integral to how the construct is conceptualised in contemporary research; a consistent finding across the included studies is that inner psychological strengths, particularly self-efficacy, spirituality, and self-esteem, predicted resilience more strongly than external resources alone [70,74,76]. Gratitude and meaning-making added further to this picture; perceived personal control predicted resilience, and gratitude showed a statistically significant indirect effect in this relationship, though the cross-sectional design of that study means we cannot be certain of the direction of influence [31]. This is consistent with broader evidence that perceived control is associated with better functional health outcomes in adulthood [76]. Spirituality consistently provided a framework for accepting life’s difficulties and holding onto meaning, including in Iran, Brazil, Thailand, and the United States [30,53,77,78]. Notably, among older adults with depression, these same resilience resources, such as grit, active and accommodative coping, self-efficacy, and spirituality, retained their protective value, remaining associated with lower depression and apathy and better quality of life [48]. This suggests that psychological resilience resources do not simply disappear in the face of clinical vulnerability.

Sense of coherence was linked to resilience over time in one longitudinal study, with social support partly explaining this link [70]. This is stronger evidence than a single cross-sectional finding, but it still cannot confirm that sense of coherence causes higher resilience. The relationship may also run the other way: people with higher resilience may develop a stronger sense of coherence over time. Until trial evidence is available, sense of coherence should be seen as a promising area for future intervention research, not yet as a confirmed treatment target. Digital literacy emerged as a newer and largely overlooked individual-level resource in this literature; older adults with stronger digital skills showed greater self-efficacy, social inclusion, and autonomy, whilst those with limited technological competence were more likely to experience social exclusion and reduced access to health information [71]. As digital life becomes increasingly challenging to decline, this gap carries growing implications for resilience in older populations.

Family relationships and social networks were among the most reliably identified protective factors across the included studies, and a recurring finding was that the quality of relationships mattered more than how many connections a person had [79]. In rural settings in particular, family networks showed especially strong ties to resilience [34]. Living with multiple chronic conditions was associated with lower resilience, though social engagement and community participation helped to support this effect to some degree [80]. Better health, higher education, economic stability, and fair access to services all created conditions in which resilience was more likely to develop and be sustained [81,82]. Early life experiences also left their mark, with formative relationships and work histories linked to stronger character identity and more stable social bonds in later life [83].

Gender differences across the included studies were inconsistent, which itself is informative: rather than one gender being more resilient, gender appears to shape the individual pathways through which resilience is expressed [84,85]. Among older women in this review, resilience was more closely tied to spiritual engagement and meaningful family relationships, whilst among older men it showed stronger links to cognitive performance and functional autonomy [65,85]. Urban and rural differences in resilience were not simply a matter of where people lived; they reflected differences in access to organised social and physical activities [37]. This is an important finding: it shifts attention away from geography as a fixed disadvantage and towards community infrastructure as something that can be improved.

### 4.3. Evidence-Based Interventions for Resilience

Whether resilience can be deliberately promoted is a question central to how the construct is recognized; a fixed trait, by definition, cannot be trained. This review identified seven studies examining structured interventions to promote resilience in older adults, and their collective findings offer preliminary but meaningful evidence that resilience is modifiable, which itself carries conceptual significance [39,40,42,68]. This evidence should be interpreted with caution: five of the seven studies were randomised controlled trials, sample sizes ranged from 20 to 178 participants, and follow-up beyond the immediate post-intervention period was reported in only two studies. The evidence base here is broader than that assembled in previous reviews, though findings remain indicative rather than conclusive and are not yet sufficient to support definitive practice or policy recommendations. Physical activity, nature-based programmes, mindfulness-based yoga, and positive thinking training all showed promising signs of enhancing resilience, with evidence drawn from older adult populations in South Korea, Japan, and Iran. Forest healing programmes combining walking and meditation produced significant resilience improvements after just four sessions, with the largest gains seen in self-regulation and interpersonal ability [42]. That significant change could occur so quickly is valuable, suggesting that brief nature-based programmes may be a practical option for community settings, though replication in larger trials is needed before firm conclusions can be drawn.

Among the intervention findings, the Japanese RCT comparing mindfulness-based yoga and structured exercise stands out: resilience mediated the reduction in minor psychiatric disorders in both activities [39]. This is one of the more practical findings to emerge from this review because it suggests that when interventions successfully build resilience, broader mental health benefits are gained, making resilience a worthwhile target, not just a secondary concern. This mediating role aligns well with broader evidence from a systematic review and meta-analysis showing that mindfulness-based interventions produce significant resilience improvements compared with control conditions [72]. Positive thinking training produced resilience and life satisfaction gains that held at two-month follow-up [68], which is an encouraging finding as it suggests that the capacity to learn and apply cognitive skills does not disappear in older age, even among adults in their sixties and beyond. A pragmatic trial combining physical activity and mindfulness in a group-based format provided early feasibility evidence [43], though the study design does not allow strong conclusions about whether the programme worked. A US RCT found that both Tai Chi Chih and health education/wellness training, when combined with standard antidepressant treatment, produced significant within-group resilience gains at three and six months, with no significant difference between the two active arms, suggesting that resilience gains here may reflect shared elements such as group participation and sustained engagement rather than the specific content of either programme [40]. One finding deserves particular attention: a web-based RCT from Taiwan found no significant resilience improvements at any time point [69]. This is an important reminder that delivering a programme online does not automatically make it effective; what is delivered, how often, and to whom are all factors that matter just as much as the delivery platform. By contrast, a technology-supported intervention combining structured exercise with intelligent, sensor-based equipment produced significant resilience gains at six months among older adults in Taiwan, with qualitative interviews suggesting that digital confidence, needs-aligned exercise content, and peer connection each played a role [41], indicating that digital delivery format alone does not determine outcomes; how the technology is integrated into meaningful activity appears to matter more.

Across all seven studies, group-based delivery was the most common format, and it is reasonable to think that the social dimension of these programmes, being with others and sharing experiences, contributed to resilience gains alongside the specific intervention content. Whether interventions should be designed differently for older men and women remains an open question. Based on what this review found about gender differences in resilience pathways, programmes for older women might consider emphasising group-based social interaction, whilst those for older men might focus more on functional autonomy and cognitive goals [84,85]. These are tentative suggestions, however, and dedicated gender-stratified trials are needed before any firm recommendations can be made. Future trials in this area need larger samples, active control conditions, agreed outcome measures, longer follow-up periods, and some attention to cost-effectiveness. Recent evidence on modifiable determinants of physical activity and sedentary behaviour in older adults across community and healthcare settings [86] may usefully inform the design of future physical-activity-based resilience interventions. There is also a clear need to include groups that have been largely absent from the intervention literature, such as the oldest-old, people living in rural areas, and older adults from culturally and linguistically diverse backgrounds.

### 4.4. Resilience Outcomes and Well-Being

Across the 42 studies included in this review, resilience showed consistent associations with quality of life, life satisfaction, mental health, and psychological well-being [72]. It was positively linked to functional autonomy, cognitive performance, and subjective well-being across diverse populations [65] and consistently associated with lower depression, reduced apathy, and less psychological distress, even among older adults with clinical conditions [48,72]. In Egypt, autonomy, environmental mastery, and personal growth together accounted for 48% of resilience variance, though the cross-sectional design prevents conclusions about which drives which [55]. Life satisfaction showed a consistent positive relationship with resilience across cultural contexts, including during COVID-19 in Hong Kong, where resilience helped protect life satisfaction under prolonged adversity [45]. Among 77,395 racially diverse older women in the United States, African American women reported the highest resilience levels, with perceived control, personal energy, and opportunities for growth as the strongest modifiable determinants, a finding that points to the value of culturally affirming and strengths-based approaches [58].

Over six years, inner strength and well-being reinforced each other, and crises were linked to growth in inner strength rather than decline [61]. Higher baseline resilience was linked to slower cognitive decline over two years [47]. Declining resilience was also more strongly linked to worsening frailty than improving resilience was linked to recovery [66]. A large Chinese cohort found that higher resilience was linked to lower all-cause, cardiovascular, and respiratory mortality, with a dose–response pattern [62]. These longitudinal findings are encouraging. They suggest resilience may be more than a psychological asset and may also relate to physical health and survival. However, longitudinal association is still not the same as proof of causation. Confounding by unmeasured health or lifestyle factors remains possible. These findings should be read as a strong rationale for further trials, not as confirmed evidence that improving resilience will improve survival.

This review’s findings carry relevance when considered against core geriatric modifiers that shape how resilience is experienced and expressed in later life. Frailty, multimorbidity, cognitive impairment, functional limitation, social isolation [87], digital exclusion, and care setting (community-dwelling versus institutionalised) each intersect with resilience in distinct ways across this evidence base, from frailty progression [66] to digital literacy as an emerging resource [71] to institutional and rural care contexts [34,37]. Resilience in the young-old (60–74 years) may draw more heavily on functional autonomy and social participation, whereas resilience in the old-old and oldest-old (80+ years) appears more closely tied to psychological and spiritual resources as physical reserves decline [28,53]. Future syntheses would benefit from systematically stratifying findings by these age subgroups and geriatric modifiers rather than treating older adulthood as a single, undifferentiated category.

### 4.5. Strengths and Limitations

This review has several strengths worth noting. Searching across six major databases, with independent dual screening and third-reviewer resolution of disagreements, alongside the inclusion of diverse study designs, enabled a synthesis that captures both the breadth of quantitative findings and the depth of lived experience that qualitative studies contribute. The inclusion of 42 empirical studies across 18 countries represents a broader evidence base than previous reviews of resilience in older adults, with geographic diversity spanning Asia, Europe, South America, North America, and Africa. Six longitudinal studies provide a more practical basis for understanding resilience as a dynamic and evolving process than cross-sectional evidence alone can offer, and seven intervention studies, including five RCTs, strengthen the foundation for preliminary practice directions. The use of the MMAT ensured systematic and transparent quality evaluation across all study designs, and the deliberate inclusion of null intervention findings reflects a commitment to balanced rather than selectively positive synthesis.

Several limitations should be kept in mind. Cross-sectional quantitative studies remain the dominant design (*n* = 21), which limits causal inference and makes it difficult to say much about how resilience develops and changes over time. Measurement heterogeneity across more than twenty instruments continues to limit cross-study comparability, and the seven intervention studies were predominantly small and short in follow-up, constraining conclusions about what works best and for how long. Certain populations remain underrepresented in this literature, including the oldest-old, people in rural and remote communities, and those living with cognitive impairment or complex multimorbidity. Restricting inclusion to English-language publications may have introduced language bias, which is a particular concern given that a large proportion of the included studies came from Asian research contexts. Whilst CINAHL, MEDLINE, and PubMed were searched using the original review’s search strategy, Scopus, Web of Science, and Cochrane CENTRAL were incorporated using a refined strategy with truncation to improve sensitivity, following recommendations received during peer review (see Section 2.1). PsycINFO, AgeLine, and Embase could not be searched, as institutional subscriptions did not extend to these databases. We recognise that these databases may hold relevant evidence, particularly psychological and gerontological literature, that this review has not captured. Unequal institutional access to specialised databases is a structural constraint shared by many research teams, particularly those outside well-resourced settings, and we believe this is worth naming openly rather than glossing over: broader, equitable access to the full range of scholarly databases would meaningfully strengthen the rigour and inclusiveness of evidence synthesis across the field, and we hope future collaborations or institutional partnerships will allow this review’s search strategy to be extended further. In addition, this review was not prospectively registered (e.g., with PROSPERO), which limits transparency regarding a priori methodological decisions, and grey literature, dissertations, and conference proceedings were not systematically searched, which may have introduced additional publication bias beyond that already discussed. Publication bias may have led to some overestimation of resilience associations, though the inclusion of one null intervention finding partially mitigates this concern. Finally, whilst the integrative review methodology is well suited to synthesising diverse forms of evidence, it does not allow statistical pooling of effect sizes, and narrative synthesis inevitably involves some degree of subjectivity in how evidence is interpreted and weighted.

### 4.6. Future Research

Despite the progress documented in this review, several important gaps remain. Longitudinal research should more systematically track how resilience shifts across older adulthood, examine its bidirectional relationships with a wider range of health outcomes, and pay closer attention to critical life transition points such as retirement, bereavement, chronic illness onset, and entry into care, where resilience may be most susceptible to change. The links identified in this review between resilience and lower mortality, slower cognitive decline, and reduced frailty are preliminary but meaningful. Replication in diverse populations and settings is needed before resilience promotion can be considered a confirmed public health priority.

Larger and adequately powered RCTs are needed to evaluate intervention efficacy, underlying mechanisms, and long-term durability. The five randomised controlled trials identified in this review had modest sample sizes and limited follow-up periods, and future trials should incorporate active control conditions, standardised outcome measures, longer follow-up, and health economic components. The null finding from the web-based RCT further underlines the importance of investigating what moderates intervention outcomes, including programme content, dose, delivery mode, and participant characteristics, so that future research can better identify for whom and under what conditions resilience interventions work.

Addressing measurement challenges, a central concern of this review, will require international collaboration to reach consensus on core resilience dimensions and develop culturally valid, age-appropriate tools. As the focus of this integrative review was on conceptual-measurement alignment, future research should extend these findings through full COSMIN psychometric evaluations of existing resilience instruments to establish robust evidence for their measurement properties across diverse older adult populations. Instruments such as the Resilience Scale for Oldest-Old Age represent genuine progress, but broader cross-cultural validation remains a priority, alongside dedicated development of measures that capture stress reactivity and recovery rather than resilience as a static attribute. Greater attention to underrepresented populations such as the oldest-old, people in rural communities, culturally diverse older adults, and those living with cognitive impairment or multimorbidity is also essential, and qualitative and mixed-methods approaches are particularly suited to capturing resilience experiences that quantitative scales alone cannot reach. The relationship between resilience and digital literacy warrants dedicated investigation, particularly as digital inequalities among older adults continue to widen. Finally, implementation science frameworks should guide efforts to embed resilience-promoting approaches within real-world systems such as primary care, community nursing, aged care, and social prescribing so that research findings are translated into practice in ways that are both equitable and sustainable.

### 4.7. Recommendations for Nursing and Allied Health Practice

These recommendations are preliminary and should be tested further before wide use in practice. Nurses and interdisciplinary care teams may consider including resilience assessment in person-centred care. This could include psychological assets such as self-efficacy, gratitude, spirituality, and sense of purpose. It could also include relational and contextual resources such as family support, social connection, and community access. Resilience promotion should be integrated within comprehensive geriatric assessment, particularly for older adults living with multimorbidity, frailty, cognitive impairment, or care dependency, rather than offered as a stand-alone psychological strategy.

In terms of measurement, the CD-RISC-10 offers a brief and validated screening option for most clinical settings, whilst the RSO may be more appropriate for adults aged 80 and above [28], a distinction worth making given the measurement heterogeneity identified across this review. These recommendations rest predominantly on cross-sectional evidence and five modest-sized RCTs and should be treated as preliminary practice directions rather than definitive guidance.

Structured physical activity, mindfulness-based yoga, nature-based programmes, positive thinking training, Tai Chi, and technology-supported exercise have each shown early promise in improving resilience among older adults [39,40,41,42,68]. Group-based delivery formats are worth prioritising where feasible, as they address social isolation at the same time as delivering intervention content [62]. Digitally delivered programmes may offer scalability, though mixed findings, including one null result, indicate that content and dose matter as much as the delivery platform [69]. The finding that resilience fully mediated psychiatric disorder reduction in both arms of a Japanese RCT suggests that targeting resilience directly, rather than treating it as a secondary concern, may expand broader mental health benefits [39].

The longitudinal evidence linking resilience to slower cognitive decline, reduced frailty, and lower mortality means that resilience promotion is relevant to physical health monitoring and care planning, not only mental health [47,62,66]. Practitioners should attend to existential, spiritual, and meaning-making dimensions as part of holistic care and resist deficit-focused approaches that examine the strengths older adults already bring [30,53,59]. Gender-sensitive and culturally adapted approaches are warranted given that gender and cultural context shape how resilience is expressed and built [84,85], and clear referral pathways to community resources such as social prescribing, digital literacy training, and mental health services should be actively maintained.

### 4.8. Recommendations for Health Policy and Systems

As ageing populations expand globally, policymakers should consider resilience promotion as a meaningful component of healthy ageing strategies. That said, the current evidence base, whilst strengthened by 42 empirical studies including six longitudinal studies and five RCTs, remains predominantly cross-sectional and is not yet sufficient to support large-scale policy mandates. Policy recommendations from this review should therefore be treated as evidence-informed directions that require stronger experimental and longitudinal support before they are adopted at scale.

At the international level, resilience promotion could be considered within the United Nations Decade of Healthy Ageing (2021–2030), which calls for coordinated global action to improve the lives of older people and the communities in which they live [2]. WHO monitoring frameworks could incorporate resilience-related indicators as the evidence base matures, though the longitudinal findings linking resilience to reduced mortality, slower cognitive decline, and reduced frailty progression identified in this review warrant replication in larger and more diverse samples before inclusion in such frameworks [88].

At the national level, governments may consider resilience screening and community-based programmes as part of healthy ageing strategies. This is based on early evidence for physical activity, mindfulness, and cognitive skill-building [39,40,42,68]. Equitable access matters, since geographic location, education, and economic security all shape resilience [81,82]. Resilience is increasingly understood as a multilevel construct shaped by social participation, equity, community infrastructure, and crisis preparedness, a framing supported by evidence from adjacent fields such as community sport systems [89], which may offer transferable insights for community-based resilience programming in ageing populations. Policymakers should also consider practical barriers to implementation, such as workforce training, funding, and service availability in under-resourced areas. There is also a risk in framing resilience purely as an individual responsibility. Some older adults face social disadvantage that cannot be solved through personal resilience alone. Policies should therefore pair resilience promotion with broader efforts to address structural disadvantage, such as income security and access to care.

## 5. Conclusions

Resilience in older adults is a multifaceted and amendable construct. Across 42 empirical studies spanning 18 countries, resilience showed consistent associations with quality of life, life satisfaction, mental health, and psychological well-being. Individual psychological assets, particularly self-efficacy, spirituality, and sense of purpose, emerged as initial resilience resources, alongside family relationships, social networks, and contextual conditions such as education, economic security, and community access. A growing number of longitudinal studies link higher resilience to lower mortality, slower cognitive decline, and slower frailty progression. This suggests resilience may matter for physical health, not only psychological well-being. However, these are observational findings, and causation cannot yet be confirmed.

Preliminary evidence from seven intervention studies, including five randomised controlled trials, supports physical activity, mindfulness-based approaches, positive thinking training, Tai Chi, and technology-supported exercise as promising intervention modalities, though sample sizes remain modest and long-term durability requires further investigation. Despite these advances, conceptual diversity and measurement heterogeneity across more than twenty instruments remain significant challenges, and the predominance of cross-sectional designs continues to limit causal inference. Current recommendations for nursing practice and health policy should therefore be regarded as evidence-informed directions rather than definitive guidance, and larger longitudinal studies and adequately powered randomised controlled trials are needed to build the robust evidence base required to translate these promising findings into effective, equitable, and sustainable practice and policy for ageing populations worldwide.

## Figures and Tables

**Table 1:** Thematic Framework Derived from Inductive Analysis.

Analytical Level	Theme	Categories	Example Codes
Conceptual	1. Conceptualisation and Measurement	Trait, process, and outcome perspectives; instrument heterogeneity; cultural and age-specific adaptation	CD-RISC (25-item, 10-item, adapted versions); Wagnild & Young RS; Brief Resilience Scale (BRS); RSO; RSOA; PRIFOR; Thai researcher-developed scale; phenomenological interviews
Individual	2. Individual Psychological Factors	Self-efficacy and perceived control; self-esteem; gratitude; spirituality and meaning-making; sense of coherence; digital literacy	Perceived agency; locus of control; grit and white matter integrity; gratitude mediation; trust in God, faith, contemplative practice; comprehensibility and manageability; technological competence and social inclusion
Relational & Contextual	3. Social and Relational Factors	Family relationships and perceived support quality; social networks; community and civic participation; spiritual and religious community engagement	Family bonds; grandchild relationships; network size and quality; organised activities; AADL participation; civic engagement; religious community belonging; faith co-presence
Relational & Contextual	4. Health and Contextual Characteristics	Health status and functional independence; education and marital status; economic security; geographic context; gender; digital access	Self-perceived health; ADL independence, multimorbidity, frailty; educational attainment; married status; pension income; financial stability; urban–rural disparity; community infrastructure access; gender-differentiated resilience pathways; digital equity; technological exclusion
Intervention	5. Interventions Promoting Resilience	Physical activity; nature-based programmes; mindfulness-based approaches; positive thinking and psychological skill-building; tai chi; technology-supported and web-based delivery	Structured exercise, RCT evidence, forest healing, walking and meditation, mindfulness-based yoga, psychiatric disorder mediation, positive thinking training, durable gains at follow-up, tai chi chih, resilience predicting depression remission, technology-supported exercise, digital confidence and peer connection, web-based multicomponent programmes, null findings noted

**Table 2:** Representative Studies by Theme.

Theme	Representative Studies	Design(s)
1. Conceptualisation and Measurement	Akatsuka & Tadaka [28]; Li & Ow [29]; Hassani et al. [30]	Quantitative cross-sectional, scale development; Quantitative cross-sectional, scale development; Qualitative
2. Individual Psychological Factors	Özkan & Akça [31]; Vlasova et al. [32]; Achepohl et al. [33]	Quantitative cross-sectional; Quantitative, neuroimaging secondary analysis; Mixed methods
3. Social and Relational Factors	McKibbin et al. [34]; Silveira et al. [35]; Li et al. [36]	Quantitative cross-sectional; Qualitative; Quantitative, moderated mediation
4. Health and Contextual Characteristics	Sigurðardóttir et al. [37]; Wister et al. [38]	Quantitative, population-based; Quantitative, cohort data
5. Interventions Promoting Resilience	Kukihara et al. [39]; Lavretsky et al. [40]; Tsai et al. [41]; Han & Koo [42]	RCT; RCT; RCT; Quasi-experimental

## Data Availability

The original contributions presented in this study are included in the article/Supplementary Material. Further inquiries can be directed to the corresponding author.

## Supplementary Materials

The following supporting information can be downloaded at: https://www.mdpi.com/article/10.3390/geriatrics11040111/s1, Section S1: Full Search Strategies by Database; Section S2: PRISMA 2020 Checklist; Section S3: Full Data Extraction Table for All Included Studies (*n* = 42); Section S4: Detailed Thematic Coding Framework, with Illustrative Examples; Table S1: MMAT Quality Assessment Scores.

## Abbreviations

The following abbreviations are used in this manuscript:

AADL	Advanced Activities of Daily Living
ADL	Activities of Daily Living
BRS	Brief Resilience Scale
BRCS	Brief Resilient Coping Scale
BPRS	Brief Psychological Resilience Scale
CD-RISC	Connor–Davidson Resilience Scale
FG	Focus Group
ISS	Inner Strength Scale
MDD	Major Depressive Disorder
MMAT	Mixed Methods Appraisal Tool
MRI	Multimorbidity Resilience Index
PRISMA	Preferred Reporting Items for Systematic Reviews and Meta-Analyses
PRIFOR	Physical Resilience Instrument for Older Adults
Quan.	Quantitative
RCT	Randomised Controlled Trial
RSOA	Resilience Scale for Older Adults
RSO	Resilience Scale for Oldest-Old Age
RS-11	Resilience Scale—German Short Version
SWLS	Satisfaction with Life Scale
TERS	Thai Elderly Resilience Scale
