# Supplementary material for: Navigating Adversity in Older Age: An Integrative Review of Contemporary Resilience Concepts and Measurement Challenges
Source: Geriatrics (Basel). 2026 Aug 20;11(4):111. doi: 10.3390/geriatrics11040111 (PMC13511988; doi:10.3390/geriatrics11040111)
Supplement: Supplementary file 1 [file geriatrics-11-00111-s001.zip › geriatrics-4380778-supplementary.pdf]

Supplementary Materials

Navigating Adversity in Older Age: An Integrative Review of Contemporary Resilience Concepts and Measurement Challenges

Chonlakarn Songsri, Pras Ramluggun and Pimwalunn Aryuwat

This file contains the following supporting information referenced in the main manuscript:

- Supplementary Section S1: Full Search Strategies by Database
- Supplementary Section S2: PRISMA 2020 Checklist
- Supplementary Section S3: Full Data Extraction Table for All Included Studies (n = 42)
- Supplementary Section S4: Detailed Thematic Coding Framework, with Illustrative Examples
- Table S1: MMAT Quality Assessment Scores

SUPPLEMENTARY SECTION S1

Full Search Strategies by Database

Core conceptual structure (all databases): (resilience OR “bounce back” OR “psychological resilience” OR “stress adaptation” OR hardiness) AND (“older adult” OR elder OR aged OR ageing OR senior OR geriatric), adapted to each platform's syntax and fields.
Date range applied in all databases: 1 January 2016 – 31 December 2025. Language limit: English. Publication types excluded via platform filters where available: reviews, editorials, commentaries, conference abstracts/proceedings.

1. PubMed

Field	Detail
**Interface**	PubMed.gov (NLM)
**Date executed**	During November 2025 – January 2026
**Fields searched**	Title/Abstract [tiab] and MeSH terms where mapped automatically by PubMed's Automatic Term Mapping
**Limits applied**	Publication date: 2016/01/01–2025/12/31; Language: English; Species: Humans
**Search string**	("resilience"[tiab] OR "resilien*"[tiab] OR "bounce back"[tiab] OR "psychological resilience"[MeSH Terms] OR "psychological resilience"[tiab] OR "stress adaptation"[tiab] OR "hardiness"[tiab]) AND ("older adult*"[tiab] OR "elder*"[tiab] OR "aged"[MeSH Terms] OR "aged"[tiab] OR "ageing"[tiab] OR "aging"[tiab] OR "senior*"[tiab] OR "geriatric*"[tiab] OR "Geriatrics"[MeSH Terms]). Filters: 2016/01/01–2025/12/31[dp]; English[lang]
**Records retrieved**	386 (per Figure 1)

2. MEDLINE

Field	Detail
**Interface**	EBSCOhost MEDLINE
**Date executed**	During November 2025 – January 2026
**Fields searched**	Title, Abstract, Subject Heading (.ti,ab,kw. in Ovid syntax)
**Limits applied**	Publication year 2016–2025; English language; Human
**Search string (Ovid syntax)**	1. (resilien* or “bounce back” or “psychological resilience” or “stress adaptation” or hardiness).ti,ab,kw. 2. exp Resilience, Psychological/ 3. 1 or 2 4. (“older adult*” or elder* or aged or ageing or aging or senior* or geriatric*).ti,ab,kw. 5. exp Aged/ or exp Geriatrics/ 6. 4 or 5 7. 3 and 6 8. limit 7 to (english language and yr=“2016-2025” and humans)
**Records retrieved**	363 (per Figure 1)

3. CINAHL

Field	Detail
**Interface**	EBSCOhost
**Date executed**	During November 2025 – January 2026
**Fields searched**	Title/Abstract (TI/AB); consistent with the original submission's untruncated strategy
**Limits applied**	Published Date: 20160101–20251231; Language: English; Exclude MEDLINE records: No
**Search string**	S1: TI (resilience OR “bounce back” OR “psychological resilience” OR “stress adaptation” OR hardiness) OR AB (resilience OR “bounce back” OR “psychological resilience” OR “stress adaptation” OR hardiness). S2: TI (“older adult” OR elder OR aged OR ageing OR senior OR geriatric) OR AB (“older adult” OR elder OR aged OR ageing OR senior OR geriatric). S3: S1 AND S2. Limiters: Published Date 20160101-20251231; English Language
**Records retrieved**	529 (per Figure 1)

4. Scopus

Field	Detail
**Interface**	Scopus (Elsevier)
**Date executed**	During May – June 2026
**Fields searched**	Title, Abstract, Keywords (TITLE-ABS-KEY)
**Limits applied**	Document type: Article, Article in Press; Language: English; Years: 2016–2025
**Search string**	TITLE-ABS-KEY (resilien* OR “bounce back” OR “psychological resilience” OR “stress adaptation” OR hardiness) AND TITLE-ABS-KEY (“older adult*” OR elder* OR aged OR ageing OR aging OR senior* OR geriatric*) AND PUBYEAR > 2015 AND PUBYEAR < 2026 AND (LIMIT-TO (LANGUAGE,“English”)) AND (LIMIT-TO (DOCTYPE,“ar”))
**Records retrieved**	660 (per Figure 1)

5. Web of Science (Core Collection)

Field	Detail
**Interface**	Clarivate Web of Science
**Date executed**	During May – June 2026
**Fields searched**	Topic (TS = Title, Abstract, Author Keywords, Keywords Plus)
**Limits applied**	Document Types: Article; Language: English; Years: 2016–2025
**Search string**	TS=(resilien* OR “bounce back” OR “psychological resilience” OR “stress adaptation” OR hardiness) AND TS=(“older adult*” OR elder* OR aged OR ageing OR aging OR senior* OR geriatric*). Refined by: Publication Years 2016-2025; Document Types Article; Languages English
**Records retrieved**	885 (per Figure 1)

6. Cochrane CENTRAL (Cochrane Library)

Field	Detail
**Interface**	Cochrane Library (Wiley)
**Date executed**	During May – June 2026
**Fields searched**	Abstract, Keyword
**Limits applied**	Publication years 2016–2025; Trials only (CENTRAL); Restrict to English via Cochrane's default language handling
**Search string**	#1 (resilien* OR "bounce back" OR "psychological resilience" OR "stress adaptation" OR hardiness):AB,KW. #2 ("older adult*" OR elder* OR aged OR ageing OR aging OR senior* OR geriatric*):AB,KW. #3 #1 AND #2, Publication Year from 2016 to 2025, in Trials
**Records retrieved**	2 (per Figure 1)

Notes on strategy evolution across the two review stages

Per Section 2.1 of the manuscript, PubMed, CINAHL, and MEDLINE were searched using the original (untruncated) strategy for consistency with the initial submission. Scopus, Web of Science, and Cochrane CENTRAL were added following peer review using a refined strategy incorporating truncation (resilien*, elder*, senior*, geriatric*) to improve sensitivity, as shown above.

Total records identified across all six databases: 2,825 (CINAHL n = 529; MEDLINE n = 363; PubMed n = 386; Scopus n = 660; Web of Science n = 885; Cochrane CENTRAL n = 2), consistent with Figure 1 of the manuscript.

SUPPLEMENTARY SECTION S2

PRISMA 2020 Checklist

Item #	Checklist item	Location in manuscript	Reported?
1	Identify the report as a systematic (integrative) review.	Title page: "An Integrative Review of Contemporary Resilience Concepts and Measurement Challenges"	Yes
2	Provide a structured summary including background, objectives, methods, results, and conclusions.	Abstract	Yes
3	Describe the rationale for the review in the context of existing knowledge.	Introduction, para. 1-3	Yes
4	Provide an explicit statement of the objective(s) or question(s) the review addresses.	Introduction, final paragraph ("The review addresses three aims…")	Yes
5	Specify the inclusion and exclusion criteria for the review.	2.1, Study selection	Yes
6	Specify all databases, registers, and other sources searched, and the dates of last search for each.	2.1; full detail in Supplementary Section S1	Partial: databases and date range (2016-2025) stated; exact per-database search execution dates confirmed in Supplementary Section S1
7	Present the full search strategy for at least one database, including filters and limits used.	2.1 (core string); full per-database strings in Supplementary Section S1	Yes
8	Specify the methods used to decide whether a study met the inclusion criteria, including number of reviewers and any automation tools.	2.1: two independent reviewers, disagreements resolved by a third reviewer	Yes
9	Specify the methods used to collect data from reports, including number of reviewers and any automation tools.	2.2 Data extraction	Yes
10a	List and define all outcomes for which data were sought.	2.1 resilience as outcome, predictor, or mediating/moderating variable	Yes

10b	List and define all other variables for which data were sought (e.g., participant and study characteristics).	2.2 demographics, resilience definitions/instruments, predictor domains	Yes
11	Specify the methods used to assess risk of bias/methodological quality in included studies, including number of reviewers.	2.3 Quality assessment (MMAT)	Yes
12	Specify the effect measure(s) used in synthesis or presentation of results.	Table 3 and Table 4 report study-specific effect sizes/statistics as extracted; no single common effect measure was pre-specified, consistent with a narrative (non-meta-analytic) synthesis	Partial: no pooled effect measure applicable
13a	Describe the process used to decide which studies were eligible for each synthesis.	2.4 Data synthesis	Yes
13b	Describe methods for preparing data for presentation or synthesis (e.g., handling of missing data).	2.2 Data extraction; 2.4 Data synthesis	Partial: general extraction process described; no explicit missing-data procedure stated
13c	Describe methods for tabulating or visually displaying results for individual studies and syntheses.	Table 1 (thematic framework), Tables 2-4, Figure 1	Yes
13d	Describe methods used to synthesize results.	2.4, narrative synthesis with thematic analysis (constant comparison)	Yes
13e	Describe methods to explore possible causes of heterogeneity among study results (e.g., subgroup analysis, meta-regression).	Not applicable, narrative/thematic synthesis; qualitative findings grouped by subgroup (2.4)	N/A for quantitative heterogeneity; subgroup grouping described for qualitative synthesis
13f	Describe any sensitivity analyses conducted to assess robustness of synthesized results.	Not applicable: no meta-analysis conducted	N/A
14	Describe methods used to assess risk of bias due to missing results (reporting bias) in each synthesis.	4.5 discusses publication bias narratively; no formal reporting-bias assessment tool applied	Partial / N/A: discussed qualitatively as a limitation, not formally assessed
15	Describe methods used to assess certainty (or confidence) in the body of evidence for an outcome.	Not applicable, GRADE or equivalent certainty-of-evidence rating not used; MMAT used for individual study quality instead	N/A
16a	Describe the results of the search and selection process, ideally with a flow diagram.	3.1; Figure 1 (PRISMA flow diagram)	Yes
16b	Cite studies that might appear eligible but were excluded, and explain why.	3.1; full list with reasons in Supplementary Section S3	Yes
17	Cite each included study and present its characteristics.	Tables 3 and 4	Yes

18	Present assessments of risk of bias/methodological quality for each included study.	3.2; Tables 3 and 4 (MMAT column); item-level detail in Table S1	Yes
19	Present results for all outcomes sought, for each study.	Tables 3 and 4, "Key Findings" and "Effect Size" columns	Yes
20a	Briefly summarize the characteristics and risk of bias among studies contributing to each synthesis.	3.3; 3.4–3.9 (narrative synthesis by theme)	Yes
20b	Present results of all statistical syntheses conducted, including heterogeneity statistics.	Not applicable - no meta-analytic pooling performed; results synthesized narratively by theme (3.4–3.9)	N/A
20c	Present results of any investigations of possible causes of heterogeneity among study results.	Not applicable	N/A
20d	Present results of any sensitivity analyses.	Not applicable	N/A
21	Present assessment of risk of bias due to missing results for each synthesis.	4.5, Strengths and Limitations, discussed narratively	Partial
22	Present assessment of certainty in the body of evidence for each outcome.	Not applicable, no formal certainty rating (e.g., GRADE) applied; overall evidence strength discussed narratively in Discussion	N/A
23a	Provide a general interpretation of results in the context of other evidence.	4.1; 4.4	Yes
23b	Discuss limitations of the evidence included in the review.	4.5, Strengths and Limitations	Yes
23c	Discuss limitations of the review processes used.	4.5, Strengths and Limitations	Yes
23d	Discuss implications of the results for practice, policy, and future research.	4.6 (Future Research), 4.7 (Nursing/Allied Health Practice), 4.8 (Health Policy and Systems)	Yes
24a	Provide registration information, including register name and registration number, or state that the review was not registered.	4.5 explicitly states the review was not prospectively registered (e.g., with PROSPERO)	Yes, reported as not registered
24b	Indicate where the review protocol can be accessed, or state that a protocol was not prepared.	Not stated in manuscript	N/A
24c	Describe and explain any amendments to information provided at registration or in the protocol.	Not applicable, no prospective registration/protocol	N/A
25	Describe sources of financial or non-financial support for the review, and the role of funders.	Funding statement, "This research received no external funding."	Yes
26	Declare any competing interests of review authors.	Conflicts of Interest statement, "The authors declare no conflicts of interest."	Yes

27	Report which data, analytic code, and other materials, if any, are publicly available and where they can be found.	Data Availability Statement, "Data are contained within the article."; extended data in Supplementary Sections S1–S4 and Table S1	Yes

Source: Checklist structure follows the PRISMA 2020 statement (Page et al., 2021). Wording of checklist items above is paraphrased for this worksheet; consult the official PRISMA 2020 checklist and explanation & elaboration paper for exact item wording if required by the target journal.

SUPPLEMENTARY SECTION S3

Full Data Extraction Table for All Included Studies (n = 42)

This table expands the summary data presented in Tables 3 and 4 of the main manuscript into a single, complete data-extraction record for all 42 included studies, organised in two parts consistent with the main text: Part A (n = 35) covers studies addressing conceptualisation, measurement, and associated factors; Part B (n = 7) covers intervention studies. Effect sizes and key findings are summarised; readers seeking full statistical detail should consult the original source articles cited in the Reference List.

Part A. Studies Addressing Conceptualisation, Measurement, and Factors Associated with Resilience (n = 35)

No	Author (Year)	Country	Study Design	Sample (n) & Age	Resilience Measure(s)	Key Findings (summary)	Effect Size	MMAT
1	Achepohl et al. (2022)	USA (California)	Mixed methods, sequential explanatory	Survey n=61 (35 online, 26 paper); Qual subsample n=12	10-item CD-RISC; PTGI-SF	High-resilience participants used long-held contemplative practices (meditation, prayer, gratitude) to adapt to COVID-19	N/A (extreme-case qualitative comparison)	4/5
2	Akatsuka & Tadaka (2021)	Japan (Yokohama)	Quantitative, cross-sectional (scale development)	n=1283 valid (of 3000), oldest-old ≥80y	RSO (9-item, 1-factor, newly developed)	CFA identified good-fitting 9-item, 1-factor RSO; correlated with PGC Morale Scale and SAS-WL	α=.800; GFI=.979, AGFI=.963, CFI=.973, RMSEA=.049; r=.492/.559 ($p<.001$)	5/5
3	Cai et al. (2021)	China (Shanghai)	Quantitative, cross-sectional	n=196 disabled elders in nursing houses, mean age 74.5±6.81	10-item CD-RISC	High resilience enhanced sleep quality; perceived stress partially mediated the effect (2-step model)	Mediation significant (coefficients not extracted)	4/5
4	Chan et al. (2022)	Hong Kong SAR, China	Mixed methods; reclassified as Qualitative	Quant n=1067 telephone survey (all ages); Qual n=10 older adults	No named scale; subjective well-being, COVID-19	Older adults less worried about COVID-19, better subjective well-	Not specified	4/5

					worry, social capital measures	being; 5 qualitative themes incl. life philosophy and coping		
5	Hassani et al. (2017)	Iran	Qualitative, descriptive phenomenology (Colaizzi's method)	n=22 hospitalised elderly with chronic disease; 24 interview sessions	N/A (qualitative)	4 themes: meaning of resilience; growth context; external factors; personal factors to overcome illness	N/A (qualitative)	5/5
6	Hu et al. (2022)	Taiwan	Quantitative, scale development/validation, cross-sectional	n=200, mean age 76.4±6.6; 51.0% women	PRIFOR (16 items, 3 factors, newly developed)	Satisfactory item fit, unidimensionality (Rasch model), promising known-group and criterion-related validity	Cronbach's α = 0.94	4/5
7	Kim et al. (2024)	South Korea	Non-randomised, prospective longitudinal cohort (KFACS)	n=1826 (of 3014), mean age 77.6±3.7	Brief Resilience Scale (BRS)	Higher baseline BRS associated with lesser 2-year decline in global cognitive function (MMSE); no association with other domains	B=0.175, 95% CI 0.025–0.325	5/5
8	Laird et al. (2019)	USA	Quantitative, cross-sectional (baseline data from 3 trials)	n=337 ≥60y with major depressive disorder	CD-RISC	EFA revealed 4-factor model (grit, active/accommodative coping self-efficacy, spirituality); associated with older age, lower cognition, higher QOL, lower depression/apathy	Correlations and GLM (specific r/β not extracted)	3/5
9	Li & Ow (2022)	Taiwan	Quantitative, scale development, cross-sectional	Main survey n=368; test-retest subsample n=76	RSOA (15 items, 4 constructs, newly developed)	Good reliability and validity; all 4 constructs significantly correlated with life satisfaction	Cronbach's α = 0.882	4/5

10	Li et al. (2025)	China (Jinan, Shandong)	Quantitative, cross-sectional (moderated mediation)	n=1758 older adults 60+	10-item CD-RISC	Social support negatively predicted cognitive frailty; resilience partially mediated; education moderated the relationship	B=−0.066 (direct, p<.01); B=−0.103 (mediation, p<.001); B=−0.184 to −0.315 by education	4/5
11	McKibbin et al. (2016)	USA (rural/frontier Wyoming)	Quantitative, cross-sectional mailed survey	Random sample of registered voters aged 60+	Resilience Scale (Wagnild-type, implied)	Family networks and mental health status predicted resilience; mental health status moderated network–resilience relationship	Family networks p=.024; mental health p<.001; moderation p=.004/.021	3/5
12	Musich et al. (2022)	USA	Quantitative, cross-sectional survey	n=3573 adults aged 65+	Resilience level (low/medium/high) + protective factors index	Prevalence low/medium/high 27%/29%/44%; higher resilience linked to higher QOL, lower healthcare utilisation/expenditure	Not extracted (regression-based associations in full text)	4/5
13	Özkan & Akça (2022)	Turkey (Malatya)	Quantitative, cross-sectional (relational screening model)	n=200 elderly at Elderly Care Center	Brief Psychological Resilience Scale; General Self-Efficacy Scale; Gratitude Scale	Positive relationship between self-efficacy, gratitude, and resilience; gratitude mediated the self-efficacy–resilience relationship	Baron & Kenny mediation with t-test (coefficients not extracted)	3/5
14	Palanbek Yavaş & Baysan (2025)	Turkey	Quantitative, methodological (cross-cultural validation), cross-sectional	n=566 individuals aged 65+	Turkish-adapted RSOA (33 items, 4 sub-dimensions)	Retained original 4-factor structure; strong convergent validity with Quality of Life in Older Adults Scale	Cronbach's α = 0.93; Guttman split-half = 0.723; r=.657 with QOL (p<.001)	4/5

15	Perez-Rojo et al. (2022)	Spain	Quantitative, cross-sectional descriptive survey	n=889 adults aged 60+	MacLeod et al. (2016) multidimensional framework (instrument not detailed)	Older age, female sex, better health, not losing a loved one to COVID-19, gratitude, personal growth, and life purpose associated with greater resilience	Not extracted (correlational/regression analyses in full text)	4/5
16	Qiu et al. (2023)	China (rural Anhui)	Quantitative, longitudinal, multilevel analysis (non-randomised)	n=884 rural older adults 60+	Residuals-based (life satisfaction and depressive symptoms regressed on adversity, not a standard scale)	43% exhibited both Type-1/Type-2 resilience; self-rated health, financial satisfaction, social networks were common predictors	Multilevel regression coefficients (not extracted)	4/5
17	Reis & Menezes (2017)	Brazil (Jequié, Bahia)	Qualitative, phenomenological (Heideggerian)	n=14 long-living older adults	N/A (qualitative)	Religiosity/spirituality served as resilience strategies for coping, health maintenance, protection, and satisfactory ageing	N/A (qualitative)	5/5
18	Rodrigues & Tavares (2021)	Brazil (Uberaba)	Quantitative, cross-sectional household survey	n=808 elderly, mostly female, aged 60–79	Resilience Scale (Wagnild & Young, Brazilian validated version)	Total score 78.06 (men 81.53; women 76.32); associated with male sex, positive self-perceived health, AADL participation, fewer morbidities	Student's t-test/multiple regression, $p<.05$ (β not extracted)	5/5
19	Sharaf et al. (2019)	Egypt (Alexandria)	Quantitative, cross-sectional	n=150 community-dwelling older adults	Resilience scale (not confirmed) & Ryff Psychological Well-Being Scale	PWB strongly correlated with resilience; significant with 5/6 PWB dimensions, weak with positive relationships	$r=.70$ (PWB); $r=.54/.53/.49/.36/.29$ by dimension; $\beta=.59$, all $p<.001$	3/5

20	Shi et al. (2025)	China (Chenzhou, Hunan)	Quantitative, cross-sectional (stratified cluster sampling)	n=266 empty-nest older adults	CD-RISC	Healthy ageing score 134.34±20.06; resilience score 72.52±13.97; living arrangement, income, social activity significant	F=25.454, p<.001	4/5
21	Sigurðardóttir et al. (2022)	Iceland (Northern)	Quantitative, cross-sectional, population-based	n=180 (105 urban, 75 rural), aged ≥65	CD-RISC	Urban dwellers had more education, better activity access, and higher resilience than rural dwellers	Urban vs rural p<.001; education p<.001; activity access p<.001; health literacy/mental health p<.001	5/5
22	Silva Júnior et al. (2019)	Brazil (Campina Grande)	Quantitative, cross-sectional	n=86 urban elderly	Wagnild & Young RS; Social Support Scale	High resilience and moderate social support; resilience significantly associated only with religion	χ^2=0.30, p=.027 (resilience–religion); p=.005 (independence–support)	3/5
23	Silveira et al. (2018)	Brazil (Belo Horizonte)	Qualitative ("signs, meanings and actions" model)	n=13 robust/pre-frail elderly, aged 69–86	N/A (qualitative)	Resilience built through bonds with family, reinvention of self after trauma, and religiosity as strength/belonging	N/A (qualitative)	5/5
24	Springfield et al. (2022)	USA	Quantitative, cross-sectional (secondary analysis)	n=77,395 women aged 62+ (WHI Extension Study)	Brief Resilience Scale (short version)	Higher resilience associated with younger age, fewer stressors, more resources; Black/African American women reported highest resilience	Race/ethnicity × energy interaction p=.0017; elastic net regression (coefficients not extracted)	3/5
25	Thongkhum et al. (2022)	Thailand	Mixed methods (sequential	Qual n=6; Quant n=310, elderly with chronic	Newly developed Thai Elderly	Resilience defined by 3 themes, 9 categories; new scale	Cronbach's α = 0.85; CFA χ^2=161.51, df=186,	4/5

			exploratory: FGD → survey)	disease & depression	Resilience Scale (21 items)	showed good item-total correlations and construct validity	p=.90, RMSEA=.000	
26	Upasen et al. (2024)	Thailand	Quantitative, cross-sectional (SEM/path analysis)	n=964 older adults (multi-stage random sampling)	Thai Elderly Resilience Scale; Social Support Scale; Thai Geriatric Mental Health Assessment	High levels of resilience, social support, mental health; social support directly and indirectly influenced mental health via resilience	Path analysis coefficients (not extracted)	4/5
27	Viglund et al. (2021)	Finland & Sweden	Longitudinal, quantitative (2 waves; non-randomised)	n=4023, born 1930/1935/1940/1945	Inner Strength Scale (ISS)	Crises/diseases positively predicted inner strength over time; inner strength and well-being had reciprocal positive relationship	Cross-lagged panel SEM path coefficients (not extracted)	4/5
28	Vlasova et al. (2018)	USA	Quantitative, cross-sectional (neuroimaging)	n=70 older adults with major depressive disorder	CD-RISC, 4-factor EFA (grit, active/accommodative coping self-efficacy, spirituality)	"Grit" factor positively associated with white matter integrity (fractional anisotropy) in genu of corpus callosum and cingulum	F(1,64)=7.54, p=.008 (genu–Grit); F(1,64)=4.47, p=.04 (cingulum–Grit)	3/5
29	Wang et al. (2024)	China	Non-randomised, prospective cohort (CLHLS)	n=4935 participants aged 65+	5-item psychological resilience measure (CLHLS)	Higher resilience independently associated with lower all-cause and cause-specific mortality (not cancer); dose–response relationship	HR=0.74 all-cause (95% CI 0.67–0.82); HR=0.74 CVD; HR=0.63 respiratory; HR=0.69 other	5/5
30	Windle et al. (2020)	Wales, UK	Mixed methods, two-stage cross-sectional (CFAS Wales cohort)	n=20 semi-structured interviews (subsample), aged 65+	Resilient status defined quantitatively via well-being relative to comorbidity	4 life-experience themes (early years, work, adverse events, caring); 4 resilience mechanisms (identity, insight,	N/A (qualitative)	4/5

						relationships, belonging)		
31	Wister et al. (2022)	Canada	Quantitative, longitudinal (non-randomised); CLSA	n=9211 older adults with 2+ chronic conditions	Multimorbidity resilience index (functional, social, psychological sub-indices)	Index inversely associated with pandemic impact and worry; psychological sub-index most consistent; functional sub-index not associated	OR = 0.83–0.89 across models (p<.001 or p<.05)	4/5
32	Wister et al. (2024)	Canada	Quantitative, longitudinal (CLSA)	n=13,064 older adults with multimorbidity, aged 65+	CD-RISC	Strongest associations: self-rated health, sleep satisfaction, appetite, household income, more relatives/friends, being overweight, fewer housing problems	Linear regression coefficients (not extracted)	4/5
33	Wosiack et al. (2018)	Brazil (Ivoti)	Quantitative, cross-sectional	n=161 elderly, both sexes	Resilience Scale (Wagnild & Young)	Resilience correlated with QOL, basic psychological needs, wisdom, cognition; inversely with depression	Spearman correlations and linear regression (coefficients not extracted)	3/5
34	Ye et al. (2024)	China (Shanghai)	Non-randomised, prospective cohort study	n=4033 community-dwelling older adults, mean age 71.0±6.1	25-item CD-RISC	Higher resilience associated with reduced frailty progression, especially among higher baseline frailty; ΔPR nonlinearly associated with improvement/deterioration	β=−0.136 (95% CI −0.214 to −0.057); OR=1.28 (improvement); OR=1.30 (ΔPR improve); OR=0.74 (ΔPR deteriorate)	5/5

35	Carta et al. (2022)	Italy (Cagliari, Sardinia)	Non-randomised (post-RCT follow-up cohort, nested case-control)	n=79 (of 105 RCT completers), mean age 72.3±4.7, 56% women	BSRS (Social/Behavioral Rhythms functioning); PHQ-9	Frequency of major depressive episode did not significantly change before vs. during lockdown; impaired SBR functioning at week 12 raised risk of depressive episode later	MDE frequency OR=2.60, 95% CI 0.87–9.13; impaired SBR→depressive episode OR=5.6, 95% CI 1.5–21.4	3/5

Part B. Intervention Studies Promoting Resilience in Older Adults (n = 7)

No	Author (Year)	Country	Study Design	Sample (n) & Age	Intervention Type	Sessions/Duration	Follow-up	Resilience Measure (s)	Key Findings & Effect Size	MMAT
1	Han & Koo (2018)	South Korea	Quasi-experimental (single group, pre-post; no control)	n=20; mean age 71.45; 65–75y; 75% female	Nature-based forest healing programme (walking + meditation, urban forest)	4 sessions; 90 min each; twice weekly	2 weeks total; no follow-up reported	KRQ-53 (53-item)	Resilience improved significantly post-programme, greatest gains in self-regulation and interpersonal ability. Pre M=168.4 vs post M=178.0, t=−4.554, p<.05	2/5
2	Kukihara et al. (2020)	Japan	RCT (three-arm: exercise, mindfulness-yoga, control)	n=55; mean age ≈70; 65+ years	Structured exercise vs. mindfulness-based yoga vs. control (TV watching)	Weekly; 50 min each	12 weeks; post-intervention only	Wagnild & Young RS (25-item; α=.96)	Both exercise and yoga significantly improved resilience vs. control; resilience fully mediated reduction in minor psychiatric disorders in both arms. F(2,54)=5.03, p=.01	4/5
3	Taherkhani et al. (2023)	Iran	RCT (parallel group: intervention vs. control)	n=100 (intervention n=50; control n=50); 60–70 years	Positive thinking training; cognitive reframing and optimistic thinking skills	8 sessions; 90 min each; weekly	8 weeks; baseline, 1-week post, 2-month follow-up	CD-RISC (25-item; α=.97)	Resilience and life satisfaction significantly higher in intervention group at post-test and 2-month follow-up. Cohen's d=2.16 (post-test); d=1.15 (2-month)	4/5
4	Treichler et al. (2020)	USA	Pragmatic modified stepped-	n=89; mean age 84.9 (SD=7.0); senior housing residents	'Raise Your Resilience' (RYR) group intervention:	3 sessions; 90 min each	1-month active; 1-month control;	CD-RISC (10-item);	Resilience and perceived stress improved significantly at 3-	3/5

			wedge trial		savoring, gratitude, value-based activities		3-month post-intervention follow-up	PSS; SF-12; SD-WISE; LOT-R	month follow-up; wisdom subscale also improved; no significant change in well-being/happiness/optimism. CD-RISC Cohen's d=0.115	
5	Wu et al. (2024)	Taiwan	Single-blind, parallel-group RCT	n=63 (intervention n=31; control n=32); mean age 72.11; 65–92y	Web-based resilience-enhancing programme (Resilience in Illness Model) via LINE platform	4 sessions; 60 min each; weekly	4 weeks; baseline (T0), 4 weeks (T1), 12 weeks (T2)	CRS (25-item); PASE; WBS	No significant between-group differences in resilience, physical activity, or well-being at any time point; feasible and acceptable but did not demonstrate efficacy. No meaningful effect sizes	3/5
6	Lavretsky et al. (2022)	USA	Randomised controlled trial (single-blind)	n=178 randomised (89 Tai Chi Chih, 89 Health Education/Wellness); 125 completed 3-mo, 117 completed 6-mo	Tai Chi Chih (TCC) vs. Health Education and Wellness training (HEW), both combined with stable antidepressant treatment	Weekly sessions, 60 min each	12 weeks active; naturalistic follow-up to 6 months	CD-RISC	Both TCC and HEW improved depression; CD-RISC improved significantly within both groups at 3 and 6 months, no significant between-group difference; TCC showed greater improvement in general health. TCC +4.71 (SD 13.25) vs HEW +3.66 (SD 9.75) at 3mo, F(1,174)=0.3, p=.6	4/5
7	Tsai et al. (2025)	Taiwan	Mixed methods (RCT	n=69 randomised from a senior fitness club;	Intelligent/smart sports equipment	Structured phase: 24 hours over 12 weeks,	6 months; baseline	Chinese version of the	Resilience scores increased significantly from	4/5

			with embedded qualitative interviews)	intervention group completed ≥6 months; n=16 qualitative subsample	(IoT, big data, AI-driven) exercise programme	embedded within overall engagement	and 6-month assessme nt	Resilienc e Scale (CRS; 6 items, translated from the Brief Resilienc e Scale)	baseline to 6 months (21.35±4.37 to 23.35±5.4, P=.023); qualitative themes: digital adaptation, needs-aligned exercise, peer relationships	

Abbreviations: AADL = Advanced Activities of Daily Living; BPRS = Brief Psychological Resilience Scale; BRS = Brief Resilience Scale; BSRS = Brief Symptom Rating Scale; CD-RISC = Connor-Davidson Resilience Scale; CFA = Confirmatory Factor Analysis; CI = Confidence Interval; CLHLS = Chinese Longitudinal Healthy Longevity Survey; CLSA = Canadian Longitudinal Study on Aging; CRS = Chinese Resilience Scale; EFA = Exploratory Factor Analysis; GLM = General Linear Model; HR = Hazard Ratio; ISS = Inner Strength Scale; KFACS = Korean Frailty and Aging Cohort Study; KRQ-53 = Korean Resilience Quotient-53; LOT-R = Life Orientation Test-Revised; MDE = Major Depressive Episode; MMAT = Mixed Methods Appraisal Tool; MMSE = Mini-Mental State Examination; OR = Odds Ratio; PASE = Physical Activity Scale for the Elderly; PGC = Philadelphia Geriatric Center (Morale Scale); PHQ-9 = Patient Health Questionnaire-9; PRIFOR = Physical Resilience Instrument for Older Adults; PSS = Perceived Stress Scale; PTGI-SF = Posttraumatic Growth Inventory-Short Form; PWB = Psychological Well-Being; QOL = Quality of Life; RCT = Randomised Controlled Trial; RSO = Resilience Scale for Oldest-Old Age; RSOA = Resilience Scale for Older Adults; SAS-WL = Self-Anchoring Scale for feeling life is Worth Living; SBR = Social and Behavioral Rhythms; SD-WISE = San Diego Wisdom Scale; SEM = Structural Equation Modelling; SF-12 = Short Form-12 Health Survey; TCC = Tai Chi Chih; WBS = Well-Being Scale; WHI = Women's Health Initiative.

SUPPLEMENTARY SECTION S4

Detailed Thematic Coding Framework, with Illustrative Examples

This file expands Table 1 of the main manuscript, providing a fuller description of how the five themes were constructed through inductive thematic analysis (Braun & Clarke, 2022) and constant comparison, and how each theme is illustrated across the included studies. Illustrative material below is paraphrased from the findings reported in the included primary studies (as synthesised in Sections 3.4–3.8 of the manuscript) rather than reproduced as direct participant quotations, since original transcripts were not available to the review team beyond what each publication itself reported.

Coding process overview

Two reviewers independently coded all 42 included studies using constant comparison. Initial codes were generated close to the data (e.g., "contemplative practice," "grandchild relationships," "urban-rural service access"), then grouped into categories, and categories were grouped into the five broader themes presented here. Disagreements in coding were resolved through discussion, with a third reviewer consulted when consensus could not be reached, consistent with the disagreement-resolution procedure used for screening, data extraction, and quality appraisal. No formal assessment of thematic saturation was undertaken, consistent with the manuscript's stated approach (2.4), as this concept is more commonly applied in primary qualitative research than in evidence synthesis.

Theme 1: Conceptualisation and Measurement
Analytical level: Conceptual
Definition: This theme captures how included studies explicitly or implicitly framed resilience, as a stable personal trait, a dynamic adaptive process, or a measurable outcome of successful coping, and the instruments used to operationalise it.
Categories: Trait, process, and outcome perspectives; instrument heterogeneity; cultural and age-specific adaptation.
Example codes: CD-RISC (25-item, 10-item, and adapted versions); Wagnild & Young Resilience Scale; Brief Resilience Scale (BRS); RSO; RSOA; PRIFOR; Thai researcher-developed scale; phenomenological interviews.
Illustrative manifestation across studies:

- Trait-oriented framing: resilience as enduring attributes such as self-efficacy, perseverance, and sense of purpose, reflected in the CD-RISC's underlying grit/coping/spirituality factor structure (Laird et al., 2019) and in purpose-built trait inventories such as the Resilience Scale for Oldest-Old age (Akatsuka & Tadaka, 2021).
- Process-oriented framing: resilience as developing through sustained engagement between the person, relationships, and environment, evident in accounts of "reinventing oneself" in older age (Silveira et al., 2018) and multidimensional functional/psychological/social resilience sub-indices (Wister et al., 2022).
- Outcome-oriented framing: resilience as maintained or recovered well-being despite chronic illness, bereavement, frailty, or population-level adversity, including stability of depressive symptoms through COVID-19 (Carta et al., 2022) and residual well-being unexplained by adversity exposure (Qiu et al., 2023).
- Culturally/age-adapted instrument development: RSO for the oldest-old in Japan (Akatsuka & Tadaka, 2021); RSOA in Taiwan and Turkey (Li & Ow, 2022; Palanbek Yavaş & Baysan, 2025); PRIFOR for hospitalised older adults in Taiwan (Hu et al., 2022); a Thai resilience scale for chronic illness and depression (Thongkhum et al., 2022).

- Dimensions beyond quantitative measurement: patience, trust in God, reinvention of self, acceptance of chronic disease, and a life philosophy reframing hardship as ordinary, surfaced in qualitative studies (Hassani et al., 2017; Reis & Menezes, 2017; Silveira et al., 2018; Windle et al., 2020; Chan et al., 2022).

Theme 2: Individual Psychological Factors
Analytical level: Individual
Definition: This theme captures internal psychological resources consistently associated with resilience, spanning self-efficacy, self-esteem, gratitude, spirituality/meaning-making, sense of coherence, and (as an emerging resource) digital literacy.
Categories: Self-efficacy and perceived control; self-esteem; gratitude; spirituality and meaning-making; sense of coherence; digital literacy.
Example codes: Perceived agency, locus of control; grit and white matter integrity; gratitude mediation; trust in God, faith, contemplative practice; comprehensibility and manageability; technological competence and social inclusion.
Illustrative manifestation across studies:

- Self-efficacy and perceived control: predicted resilience among 200 older adults in Turkey, with gratitude showing a significant indirect effect (Özkan & Akça, 2022); perceived control, personal energy, and growth were the most influential modifiable determinants among 77,395 US women (Springfield et al., 2022).
- Gratitude, spirituality, meaning-making: contemplative practices (meditation, gratitude) sustained equanimity during COVID-19 in the US (Achepohl et al., 2022); spirituality and trust in God supported coping with chronic illness in Iran (Hassani et al., 2017); faith provided existential "co-presence" among the oldest-old in Brazil (Reis & Menezes, 2017); gratitude and life purpose were strongest predictors under pandemic stress in Spain (Perez-Rojo et al., 2022).
- Sense of coherence: identified as a longitudinal predictor of resilience trajectories, partly mediated by social support over time (Rohner et al., 2023).
- Resilience resources under clinical vulnerability: grit, active/accommodative coping self-efficacy, and spirituality remained negatively associated with depression/apathy and positively with quality of life among depressed older adults (Laird et al., 2019).
- Digital literacy (emerging resource): positively associated with self-efficacy, social inclusion, and autonomy among community-dwelling older adults in China; limited competence linked to social exclusion and reduced health-information access (Zhu et al., 2025).

Theme 3: Social and Relational Factors
Analytical level: Relational & Contextual
Definition: This theme captures how family relationships, broader social networks, community participation, and spiritual/religious community engagement function as protective, relational resources for resilience.
Categories: Family relationships and perceived support quality; social networks; community and civic participation; spiritual and religious community engagement.
Example codes: Family bonds, grandchild relationships; network size and quality; organised activities, AADL participation, civic engagement; religious community belonging, faith co-presence.
Illustrative manifestation across studies:

- Family relationships: family networks and mental health status were the strongest resilience predictors in rural Wyoming, USA, with family support buffering poor mental health (McKibbin et al., 2016); grandchild relationships provided essential meaning during frailty among the oldest-old in Brazil (Silveira et al., 2018).

- Social support as a pathway resource: partly mediated the relationship between sense of coherence and resilience over time (Rohner et al., 2023); had a significant negative effect on cognitive frailty among 1,758 older adults in China, partially mediated by resilience and moderated by education (Li et al., 2025); partially offset the adverse effects of multimorbidity on resilience in a large Canadian cohort (Wister et al., 2024).
- Spiritual/religious community engagement: patience, hope, and spirituality supported acceptance of chronic illness in Iran (Hassani et al., 2017); faith mitigated existential isolation among the oldest-old in Brazil (Reis & Menezes, 2017); religion and family support were primary external dependencies in Thailand (Thongkhum et al., 2022); internal determination and faith outweighed objective social support levels in urban Brazil (Silva Júnior et al., 2019).
- Community participation: urban older adults in Iceland showed higher resilience than rural counterparts, attributable to differential access to organised activities rather than location itself (Sigurðardóttir et al., 2022); participation in Advanced Activities of Daily Living was associated with higher resilience in Brazil (Rodrigues & Tavares, 2021); pension income, life philosophy, and telecommunications adoption buffered pandemic stressors in Hong Kong (Chan et al., 2022).

Theme 4: Health and Contextual Characteristics
Analytical level: Relational & Contextual
Definition: This theme captures health status, functional independence, and structural/contextual conditions, education, marital status, economic security, geography, gender, digital access, that shape the conditions under which resilience develops.
Categories: Health status and functional independence; education and marital status; economic security; geographic context; gender; digital access.
Example codes: Self-perceived health, ADL independence, multimorbidity, frailty; educational attainment, married status; pension income, financial stability; urban-rural disparity, community infrastructure access; gender-differentiated resilience pathways; digital equity, technological exclusion.
Illustrative manifestation across studies:

- Health status and functional independence: living at home without long-term care needs was linked to higher resilience among the oldest-old in Japan (Akatsuka & Tadaka, 2021); higher resilience enhanced sleep quality among disabled elders in Chinese nursing homes, partially mediated by perceived stress (Cai et al., 2021); resilience was linked to lower healthcare utilisation and expenditure among 3,573 US older adults (Musich et al., 2022).
- Education, marital status, economic security: financial security bolstered resilience during COVID-19 in Hong Kong (Chan et al., 2022); living arrangement, pension income, and social-activity frequency were significant correlates of healthy ageing alongside resilience in China (Shi et al., 2025).
- Gender: associations were inconsistent, female gender was linked to higher resilience in Spain (Perez-Rojo et al., 2022), while male gender was linked to higher resilience in Brazil (Rodrigues & Tavares, 2021), suggesting gender shapes pathways of expression rather than determining resilience capacity.
- Geographic context and digital access: urban-rural differences in resilience in Iceland were attributable to differential activity access, not geography itself (Sigurðardóttir et al., 2022); digital skills were positively associated with self-efficacy, inclusion, and information access, while limited competence was linked to exclusion (Zhu et al., 2025).

Theme 5: Interventions Promoting Resilience
Analytical level: Intervention
Definition: This theme captures structured programmes designed to promote resilience, spanning physical activity, nature-based programmes, mindfulness-based approaches, positive thinking/psychological skill-building, Tai Chi, and technology-supported or web-based delivery.
Categories: Physical activity; nature-based programmes; mindfulness-based approaches; positive thinking and psychological skill-building; Tai Chi; technology-supported and web-based delivery.
Example codes: Structured exercise, RCT evidence; forest healing, walking and meditation; mindfulness-based yoga, psychiatric disorder mediation; positive thinking training, durable gains at follow-up; Tai Chi Chih, resilience predicting depression remission; technology-supported exercise, digital confidence and peer connection; web-based multicomponent programmes, null findings noted.
Illustrative manifestation across studies:

- Nature-based and physical activity interventions: a quasi-experimental forest-healing programme (walking + meditation) significantly improved resilience after just four sessions in South Korea, with the largest gains in self-regulation and interpersonal ability (Han & Koo, 2018).
- Mindfulness-based approaches: an RCT in Japan found both structured exercise and mindfulness-based yoga significantly improved resilience versus control, with resilience fully mediating reductions in minor psychiatric disorders in both active arms (Kukihara et al., 2020).
- Positive thinking / psychological skill-building: an eight-week programme in Iran produced resilience and life-satisfaction gains sustained at two-month follow-up (Taherkhani et al., 2023); a US pragmatic trial using savoring, gratitude, and value-based activities showed significant but modest resilience gains at three-month follow-up (Treichler et al., 2020).
- Tai Chi: a US RCT found Tai Chi Chih and health education/wellness training, both combined with antidepressants, produced significant within-group resilience gains at three and six months, with no significant between-group difference (Lavretsky et al., 2022).
- Technology-supported and web-based delivery: an intelligent, sensor-based exercise programme in Taiwan produced significant six-month resilience gains, with qualitative themes of digital confidence, needs-aligned exercise, and peer connection (Tsai et al., 2025); by contrast, a web-based RCT (also in Taiwan) found no significant resilience improvements at any time point, underscoring that digital delivery alone does not guarantee effectiveness (Wu et al., 2024).

Cross-theme note on interpretation

The five themes are not mutually exclusive: several studies contributed evidence to more than one theme (e.g., Chan et al., 2022 informs both conceptualisation/measurement and social/relational factors; Wister et al., 2024 informs both social/relational factors and health/contextual characteristics). Table 2 of the main manuscript lists representative studies illustrating each theme; this section provides additional illustrative depth for readers seeking to understand how the coding framework was applied across the evidence base.

Table S1

MMAT Quality Assessment Scores: All 42 Included Studies

This table presents item-level Mixed Methods Appraisal Tool (MMAT, 2018 version) ratings for all 42 included studies, expanding the summary MMAT scores reported in Tables 3 and 4 of the main manuscript. Studies are grouped by MMAT design category (qualitative, mixed methods, quantitative randomised controlled trial, quantitative non-randomised, and quantitative descriptive). Numbers in the "No" column refer to each study's position in the master list of 42 included studies (see Supplementary Section S3).

Note: Y = Yes; N* = No/Cannot tell. Quality categories: High = all five criteria met (5/5); Moderate = four criteria met (4/5); Low = three or fewer criteria met (≤3/5).

Qualitative studies

Criteria: 1.1 Approach appropriate for RQ; 1.2 Data collection methods adequate; 1.3 Findings adequately derived from data; 1.4 Interpretation substantiated by data; 1.5 Coherence between sources/collection/analysis/interpretation.

No	Author (Year)	Design type	C1	C2	C3	C4	C5	Overall score
4	Chan et al. (2022)	Qualitative	Y	Y	Y	N*	Y	4/5 - Moderate*
5	Hassani et al. (2017)	Qualitative	Y	Y	Y	Y	Y	5/5 - High
17	Reis & Menezes (2017)	Qualitative	Y	Y	Y	Y	Y	5/5 - High
23	Silveira et al. (2018)	Qualitative	Y	Y	Y	Y	Y	5/5 - High

Mixed methods studies

Criteria: 5.1 Adequate rationale for mixed methods; 5.2 Effective integration of components; 5.3 Outputs of integration adequately interpreted; 5.4 Divergences/inconsistencies addressed; 5.5 Adherence to quality criteria of each tradition.

No	Author (Year)	Design type	C1	C2	C3	C4	C5	Overall score
1	Achepohl et al. (2022)	Mixed methods	Y	Y	Y	N*	Y	4/5 - Moderate*
25	Thongkhum et al. (2022)	Mixed methods	Y	Y	Y	N*	Y	4/5 - Moderate*
30	Windle et al. (2020)	Mixed methods	Y	Y	Y	N*	Y	4/5 - Moderate*

Quantitative randomised controlled trials

Criteria: 2.1 Randomization performed; 2.2 Groups comparable at baseline; 2.3 Complete outcome data; 2.4 Outcome assessors blinded; 2.5 Participants adhered.

No	Author (Year)	Design type	C1	C2	C3	C4	C5	Overall score
36	Kukihara et al. (2020)	Quantitative RCT	Y	Y	Y	N*	Y	4/5 - Moderate*
37	Taherkhani et al. (2023)	Quantitative RCT	Y	Y	Y	N*	Y	4/5 - Moderate*
39	Wu et al. (2024)	Quantitative RCT	Y	N*	Y	Y	N*	3/5 - Low*
40	Lavretsky et al. (2022)	Quantitative RCT	Y	Y	N*	Y	Y	4/5 - Moderate*
41	Tsai et al. (2025)	Quantitative RCT	N*	Y	Y	Y	Y	4/5 - Moderate*

Quantitative non-randomised studies

Criteria: 3.1 Participants representative; 3.2 Measurements appropriate; 3.3 Complete outcome data; 3.4 Confounders accounted for; 3.5 Intervention/exposure consistent.

No	Author (Year)	Design type	C1	C2	C3	C4	C5	Overall score
7	Kim et al. (2024)	Quantitative non-randomised	Y	Y	Y	Y	Y	5/5 - High
16	Qiu et al. (2023)	Quantitative non-randomised	N*	Y	Y	Y	Y	4/5 - Moderate*
27	Viglund et al. (2021)	Quantitative non-randomised	N*	Y	Y	Y	Y	4/5 - Moderate*
29	Wang et al. (2024)	Quantitative non-randomised	Y	Y	Y	Y	Y	5/5 - High
31	Wister et al. (2022)	Quantitative non-randomised	Y	Y	Y	N*	Y	4/5 - Moderate*
34	Ye et al. (2024)	Quantitative non-randomised	Y	Y	Y	Y	Y	5/5 - High
35	Han & Koo (2018)	Quantitative non-randomised	N*	Y	Y	N*	N*	2/5 - Low
38	Treichler et al. (2020)	Quantitative non-randomised	N*	Y	Y	N*	Y	3/5 - Low*
42	Carta et al. (2022)	Quantitative non-randomised	N*	Y	N*	Y	Y	3/5 - Low*

Quantitative descriptive studies

Criteria: 4.1 Relevant sampling strategy; 4.2 Sample representative; 4.3 Measurements appropriate; 4.4 Low nonresponse bias; 4.5 Appropriate statistical analysis.

No	Author (Year)	Design type	C1	C2	C3	C4	C5	Overall score
2	Akatsuka & Tadaka (2021)	Quantitative descriptive	Y	Y	Y	Y	Y	5/5 - High
3	Cai et al. (2021)	Quantitative descriptive	Y	N*	Y	Y	Y	4/5 - Moderate*
6	Hu et al. (2022)	Quantitative descriptive	Y	N*	Y	Y	Y	4/5 - Moderate*
8	Laird et al. (2019)	Quantitative descriptive	Y	N*	N*	Y	Y	3/5 - Low*
9	Li & Ow (2022)	Quantitative descriptive	Y	N*	Y	Y	Y	4/5 - Moderate*
10	Li, Ma, Wei, Wu, Cui & Zhou (2025)	Quantitative descriptive	Y	N*	Y	Y	Y	4/5 - Moderate*
11	McKibbin et al. (2016)	Quantitative descriptive	Y	N*	Y	N*	Y	3/5 - Low*
12	Musich et al. (2022)	Quantitative descriptive	Y	N*	Y	Y	Y	4/5 - Moderate*
13	Özkan & Akça (2022)	Quantitative descriptive	Y	N*	Y	N*	Y	3/5 - Low*
14	Palanbek Yavaş & Baysan (2025)	Quantitative descriptive	Y	N*	Y	Y	Y	4/5 - Moderate*
15	Perez-Rojo et al. (2022)	Quantitative descriptive	Y	N*	Y	Y	Y	4/5 - Moderate*
18	Rodrigues & Tavares (2021)	Quantitative descriptive	Y	Y	Y	Y	Y	5/5 - High
19	Sharaf et al. (2019)	Quantitative descriptive	Y	N*	Y	N*	Y	3/5 - Low*
20	Shi et al. (2025)	Quantitative descriptive	Y	N*	Y	Y	Y	4/5 - Moderate*
21	Sigurðardóttir et al. (2022)	Quantitative descriptive	Y	Y	Y	Y	Y	5/5 - High
22	Silva Júnior et al. (2019)	Quantitative descriptive	Y	N*	Y	N*	Y	3/5 - Low*
24	Springfield et al. (2022)	Quantitative descriptive	Y	Y	N*	N*	Y	3/5 - Low*
26	Upasen et al. (2024)	Quantitative descriptive	Y	Y	Y	N*	Y	4/5 - Moderate*
28	Vlasova et al. (2018)	Quantitative descriptive	Y	N*	Y	N*	Y	3/5 - Low*
32	Wister et al. (2024)	Quantitative descriptive	Y	Y	N*	Y	Y	4/5 - Moderate*
33	Wosiack et al. (2018)	Quantitative descriptive	Y	N*	Y	N*	Y	3/5 - Low*
